# Bioorthogonal Chemistry in Biomolecule Quantification: A Review of Reactions and Strategies

**DOI:** 10.1002/chem.202502315

**Published:** 2025-11-29

**Authors:** Mingze Yang, Shiqi Wang

**Affiliations:** ^1^ Institute of Biotechnology Helsinki Institute of Life Science University of Helsinki Helsinki Finland; ^2^ Faculty of Pharmacy University of Helsinki Helsinki Finland

**Keywords:** bioorthogonal chemistry, biomolecules labeling, biomolecules quantification, fluorescence imaging

## Abstract

Bioorthogonal chemistry has emerged as a transformative strategy for detecting and quantifying biomolecules in complex biological systems. This review highlights recent advances in catalyst‐free bioorthogonal reactions specifically applied to semi‐quantitative and quantitative biomolecular analysis. We exclude reactions that require toxic or complex catalysts and focus on four reactions: Staudinger ligation, strain‐promoted azide–alkyne cycloaddition, inverse electron‐demand Diels–Alder reaction, and 2‐cyanobenzothiazole–cysteine condensation. For each, we discuss reaction kinetics and strategies for representative applications in biomolecular quantification. The scope of target biomolecules varies by reaction, including proteins, nucleic acids, glycans, and small molecules. Quantification techniques such as fluorescence spectroscopy, luminescence spectroscopy, and mass spectrometry are examined, with reported limits of detection typically ranging from nanomolar to micromolar, and a few advanced techniques reaching femtomolar or attomolar sensitivity. Each reaction is discussed in terms of kinetics, molecular compatibility, and analytical sensitivity. Finally, we outline key challenges and future opportunities, emphasizing the need for faster reaction kinetics, improved probe design, enhanced integration with advanced analytical platforms, and standardized methods to improve reproducibility and cross‐study comparability in biomolecular quantification.

## Graphical abstract

This review summarizes the application of catalyst‐free bioorthogonal reactions: Staudinger ligation, strain promoted azide alkyne cycloaddition, inverse electron‐demand Diels–Alder, and 2‐cyanobenzothiazole cysteine condensation in the semi‐quantitative and quantitative analysis of biomolecules. These reactions offer fast kinetics, high selectivity, and good biocompatibility, enabling diverse detection strategies for accurate biomolecule quantification.

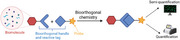

## 1 Introduction

Biomolecules, including small molecules such as metabolites and larger macromolecules like proteins and nucleic acids, play central roles in cellular processes and physiological functions [1, 2]. Accurate quantification of biomolecules is essential in molecular biology, diagnostics, and biomedical research [3, 4]. Whether measuring protein levels, nucleic acid concentrations, or metabolite abundance, quantitative analysis allows researchers to understand dynamic biological processes, monitor disease progression, and assess therapeutic outcomes. For instance, quantifying prostate‐specific antigen (PSA) levels in blood is routinely used to detect prostate cancer early, monitor disease progression, and evaluate response to treatment [5]. Quantitative detection of viral RNA, such as SARS‐CoV‐2, allows precise monitoring of viral load to evaluate infection severity and treatment efficacy [6].

Techniques such as fluorescence confocal microscopy, luminescence spectroscopy, and positron emission tomography (PET) have been developed to determine biomolecule levels either in a semi‐quantitative manner by evaluating their distribution and relative abundance or in a fully quantitative way by measuring absolute concentrations [7, 8]. These techniques typically rely on detectable signals such as fluorescence, luminescence, or radioactivity, which are not naturally present in most biomolecules. Therefore, chemical modifications of specific labels are required to introduce these signals. Such modifications demand high selectivity toward the target biomolecules, efficient performance in aqueous solutions at physiological pH, rapid reaction rates under mild conditions, and selectively labeling in complex biological system [9, 10]. Achieving all these requirements remains a significant challenge.

Bioorthogonal chemistry offers a complementary and increasingly powerful strategy for biomolecule labeling and quantification [11, 12]. These reactions are defined by their ability to proceed selectively and efficiently under physiological conditions without interfering with native biochemical processes [13, 14]. Over the past two decades, several classes of catalyst‐free bioorthogonal reactions have been developed, with the most prominent including the Staudinger ligation, strain‐promoted azide–alkyne cycloaddition (SPAAC), inverse electron‐demand Diels–Alder (IEDDA) reaction, and 2‐cyanobenzothiazole–cysteine (CBT–Cys) condensation [15, 16]. Each of these reactions offers distinct advantages in terms of kinetics, selectivity, and compatibility with biomolecular targets. With the development in the design of bioorthogonal probes and reaction conditions, their integration with analytical platforms holds growing potential for enabling quantitative biomolecule analysis under native or near‐native conditions [14].

While various bioorthogonal reactions have been extensively applied in labeling, imaging, and diagnostics, their use in biomolecule quantitative analysis was less explored. Bioorthogonal chemistry, however, is particularly well suited for quantitative analysis. These reactions typically follow second‐order kinetics, but when one reactant is present in large excess—a common case in biomolecule quantification—the kinetics approximate a pseudo–first‐order rate law, resulting in shorter half‐lives that are advantageous for generating detectable signals [17, 18]. Meanwhile, bioorthogonal handle functional groups can be readily introduced into biomolecules either through metabolic labeling—for example, azide‐labeled glycans generated by azide sugar metabolism for Staudinger ligation or SPAAC—or by exploiting naturally occurring or genetically encoded functionalities, such as N‐terminal cysteine for CBT–Cys reactions or expressed trans‐cyclooctene on proteins [19, 20]. In addition, the high selectivity of bioorthogonal reactions enables the quantification of target biomolecules without interference from complex biological environments, like the [^18^F] conjugated DIBO selectively targeted the azide antibodies [21]. Recent studies have shown that these reactions can be adapted for both semi‐quantitative and fully quantitative purposes, especially when integrated with sensitive detection platforms. For instance, Liang et al. [22]. reported a Staudinger ligation‐based method for quantifying glucose uptake in live cells. Ma et al. [23] developed a SPAAC‐based strategy to quantify miRNA. Wang et al. [24]. introduced a CBT–Cys reaction based BioLure assay for quantifying proteins delivered in live cells. These examples highlight the potential of bioorthogonal chemistry for precise molecular quantification, emphasizing the need for a comprehensive review of its applications in both semi‐quantitative and quantitative biomolecule analysis.

**FIGURE 1:**
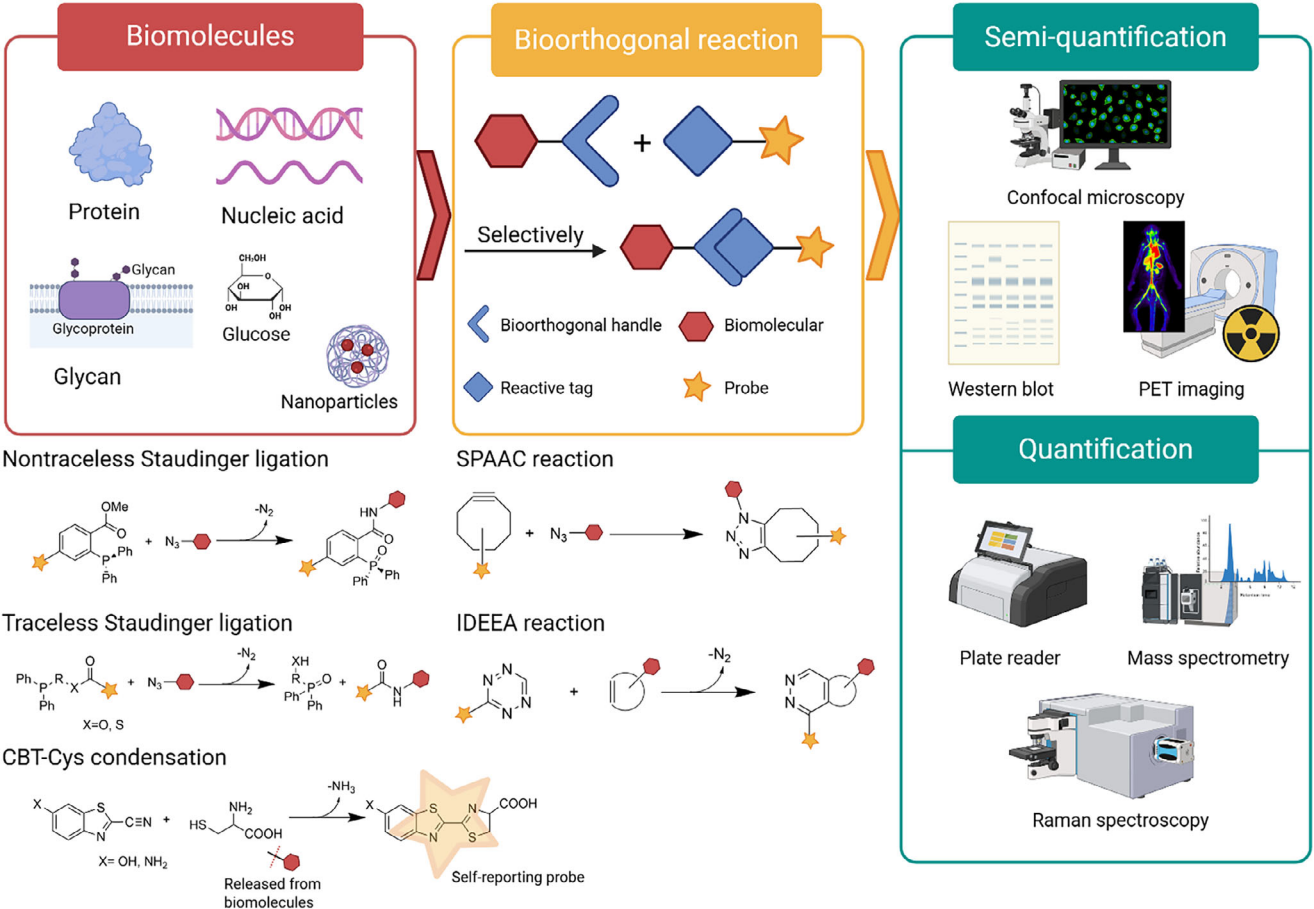
Catalyst‐free bioorthogonal reaction in the semi‐quantitative and quantitative analysis of biomolecules. Created by Biorender.com.

In this review, we present recent developments in catalyst‐free bioorthogonal reactions for the quantitative analysis of biomolecules and summarize the strengths and limitations of each reaction type (Figure 1). Reactions that require a catalyst, such as copper‐catalyzed azide alkyne cycloaddition (CUAAC), palladium‐catalyzed click reactions, oxime ligation, and photocatalyzed bioorthogonal reactions, are not covered here, as they often exhibit cellular toxicity or increase the complexity of the system [25]. Specifically, Sections 2, 3, 4, 5 focus on four catalyst‐free bioorthogonal reactions: Staudinger ligation, SPAAC, IEDDA, and CBT–Cys condensation. Each section begins with a brief overview of the reaction mechanism and kinetics. This is followed by the application of the reaction in semi‐quantitative or fully quantitative analyses, along with their corresponding detection techniques and modification strategies. Semi‐quantitative analysis aims to assess the relative content, abundance, or spatial distribution of target biomolecules, while quantitative analysis focuses on accurately measuring the absolute amount of target molecules, typically reported with standard curves and limit of detection (LOD). In Section 6, we discussed each reaction in terms of kinetics, molecular compatibility, and analytical sensitivity. Finally, the conclusion highlights the future potential of bioorthogonal chemistry in biomolecular quantification and outlines key requirements for advancing this field.

## 2 Staudinger Ligation

### 2.1 Overview

The Staudinger ligation is derived from the traditional Staudinger reaction first studied by Hermann Staudinger in 1919 [26]. It involves a bioconjugation reaction between an azide and a phosphine or phosphite resulting in covalent bond formation and can be classified into nontraceless and traceless types, depending on whether a phosphine oxide moiety remains in the final product [27]. Additionally, the Staudinger phosphite reaction is another variant of Staudinger ligation based on the phosphite substrate used. The Staudinger ligation is recognized as one of the earliest bioorthogonal reactions with excellent biocompatibility, allowing ligations both in vitro and in vivo [27, 28]. Osborn and his coworkers [29] introduced the Staudinger reaction in a previous review. Here, we briefly summarize its reaction mechanism, kinetics, and applications in biomolecules quantification.

**SCHEME 1:**
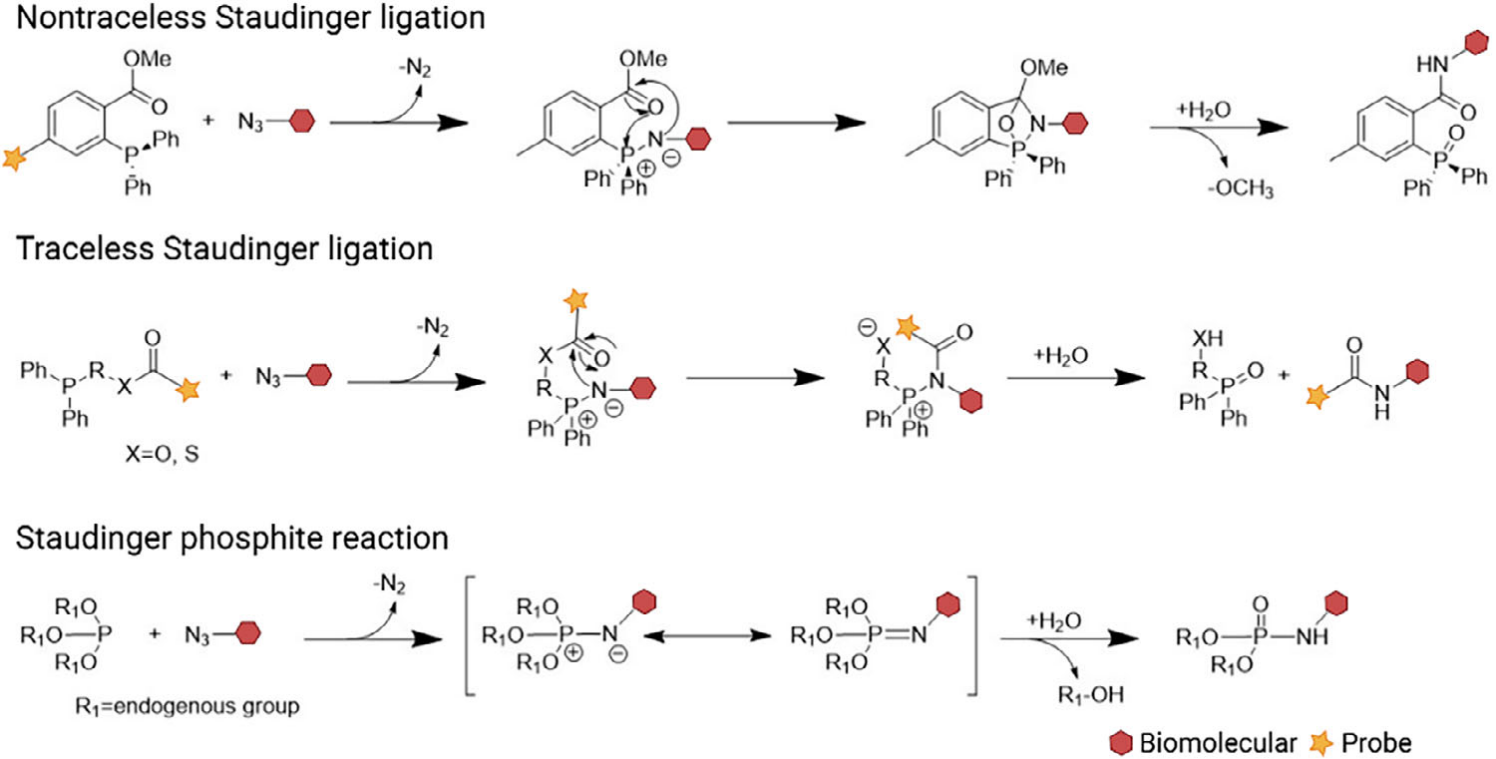
The mechanisms of three variants of the Staudinger Ligation. Adapted from Reference [30] with permission. Copyright 2020, Elsevier.

Mechanistically, the Staudinger ligation proceeds through three different pathways corresponding to its three major variants (Scheme 1.) [30]. All begin with the classical Staudinger reaction, in which an azide reacts with a phosphine or phosphite to form an iminophosphorane intermediate, accompanied by the release of nitrogen gas. In the nontraceless Staudinger ligation, the phosphine carries an electrophilic trap such as a methyl ester. The negatively charged nitrogen in the intermediate attacks the electrophilic carbon, triggering intramolecular cyclization. Subsequent hydrolysis cleaves the ring and removes the methoxy group, yielding the ligation product with a retained phosphine oxide moiety. In the traceless variant, the electrophilic trap is connected to a heteroatom (e.g., oxygen or sulfur) rather than to an aryl ring. This design enables cleavage of the bond during cyclization, releasing the phosphine oxide and yielding a product without residual phosphorus. In the Staudinger phosphite reaction, phosphite replaces phosphine and no electrophilic trap is required. Hydrolysis of the intermediate yields phosphine oxide and an alcohol byproduct.

The Staudinger ligation follows second‐order reaction kinetics, with typical rate constants on the order of 10^−^^3^ M^−^^1^ s^−^^1^ for all three variants [31]. Under equimolar conditions (10 µM each reactant), such a rate constant results in an estimated half‐life of several years [12]. Even when one reactant is present at a 100‐fold excess (1 mM), the reaction remains slow, with an estimated half‐life of approximately one week. This timescale is significantly longer than the lifetimes of most biomolecules—typically hours to days—and only comparable to long‐lived proteins, which can persist for years [32]. The reaction rate is influenced by both electronic and steric effects [33]. In general, electron‐donating substituents on the phosphine or phosphite increase the reaction rate by stabilizing the transition state, while smaller leaving groups reduce steric hindrance and further accelerate the reaction. Staudinger ligation exhibits high selectivity toward azide groups even under in vivo conditions; however, its overall selectivity can be compromised by competing side reactions and the susceptibility of phosphines to oxidation [34, 35]. From a probe design perspective, azide groups can be readily introduced on many biomolecules, including glycans, proteins, and nucleic acids via metabolic labelling or post‐modification. The azide group is stable across a wide pH range and biological redox conditions, remaining compatible with CuAAC reagents except in the presence of strong reducing agents like tris(2‐carboxyethyl)phosphine(TCEP) [36]. Meanwhile, phosphines can be functionalized with reporter groups such as fluorophores or biotin. Owing to these mechanistic and kinetic characteristics, the Staudinger ligation has been adapted for various bioorthogonal labeling strategies and even for quantitative applications under certain conditions. However, the utility of Staudinger ligation in quantitative analysis is limited by certain drawbacks, including side reactions and a lower reaction rate compared to other click reactions [35].

### 2.2 Application in Semi‐Quantitative Analysis

**FIGURE 2:**
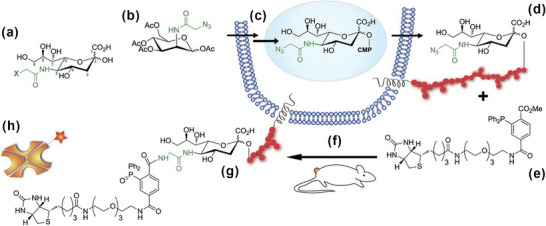
Schematic illustration of labelling and semi‐quantitative analysis of glycans on the surface of tumor cells via Staudinger ligation. Tumor glycan sialic acid (A) was metabolically labeled in living mice by intraperitoneal injection of peracetylated Ac4ManNAz (B). Ac4ManNAz is transported into cells by passive diffusion and deacetylated by intracellular carboxyesterases. ManNAz is then converted into Neu5Az in the cytosol and transported to the nucleus, where it is coupled to its carrier, cytidine monophosphate (CMP; C). Neu5Az is then incorporated into glycans by sialyltransferases in the Golgi and later transported by the ER to the tumor cell surface (D). A biotinylated phosphine bPhp (E), injected intraperitoneally (F), reacts specifically with the azido‐labeled sialic acid in the cell surface glycans (G). bPhp was detected by subsequent intravenous injection of a fluorescent or radionuclide‐labeled, neutral, and deglycosylated avidin (H).Reprinted from reference [38] with permission. Copyright 2011, Wiley.

The Staudinger ligation has been widely applied for semi‐quantitative analysis, typically in combination with probe molecules and corresponding detection techniques. Biotin is a commonly used modifier that can be easily conjugated to phosphines and subsequently applied in the semi‐quantification of azide‐modified biomolecules using fluorescence detection or western blotting. In 2007, Hang et al. [37]. reported a method using biotinylated phosphine to rapidly detect fatty‐acylated proteins in mammalian cells. During cell incubation, proteins were metabolically labeled with ω‐azido‐fatty acids and subsequently reacted with biotinylated phosphine via the nontraceless Staudinger reaction to form biotin‐tagged proteins. These proteins were then visualized by western blot using streptavidin, and the intensity or size of the blotting spots showed a positive correlation with the amount of biotin‐tagged protein. In 2011, Neves et al. [38]. reported a method employing Staudinger ligation to semi‐quantify glycans on the surface of tumor cells (Figure 2). In this approach, cell‐surface glycans were metabolically labeled with N‐azidoacetyl mannosamine and subsequently tagged with a biotinylated phosphine (bPhp) via the nontraceless Staudinger reaction. The biotin‐tagged glycans were then detected using fluorescent‐ or radionuclide‐labeled avidin via the strong biotin–avidin interaction. The detected signal intensity was positively correlated with glycan abundance on the cell surface. In addition, western blotting was also employed as a semi‐quantitative method, in which the relative size of the detected spots reflected the amount of biotin‐labeled glycans. Similarly, in 2018, Sabale et al. [39]. reported a method that utilized bioorthogonal reactions, including nontraceless Staudinger ligation, to functionalize peptide nucleic acids (PNAs) with biotinylated molecules. In this approach, PNA—capable of binding specific nucleic acid sequences—was modified with biotin and semi‐quantified using fluorescence or western blot analysis. However, a limitation of the Staudinger ligation in this work is that the yield of the labeled product is low, which can affect the sensitivity and efficiency of detection.

**FIGURE 3:**
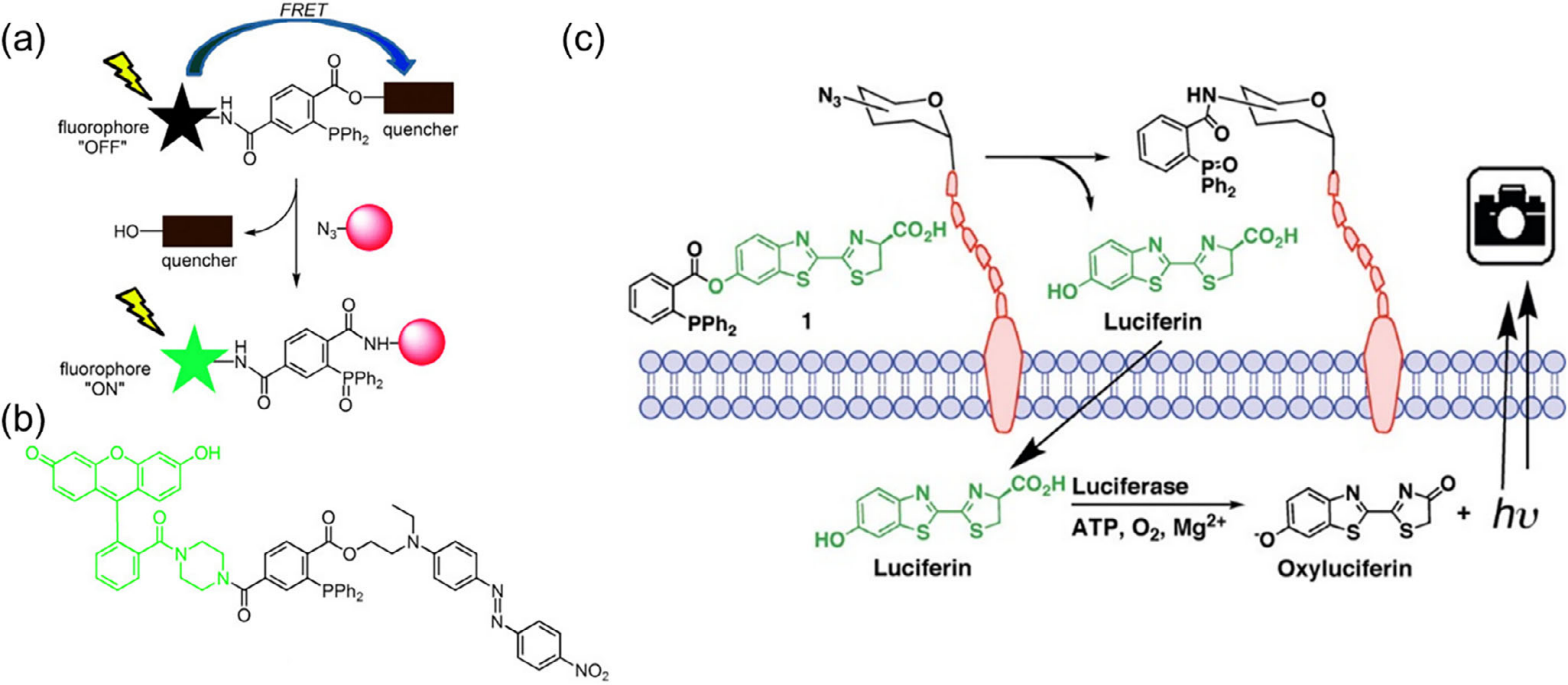
(a) The schematic illustration of the FRET fluorogenic phosphine for live‐cell imaging. (b) Structure of the designed probe, possessing fluorescein (green), and disperse red (dark red) moieties. (c) The schematic illustration of bioluminescence imaging of cell‐surface azidosugars with caged‐ luciferin phosphine reagent **1**. Adapted from References [42] and [44] with permission. Copyright 2008, Wiley.

Fluorophores represent another type of probe molecules for semi‐quantitative analysis [40]. When conjugated to phosphines or azides precursors, their intrinsic fluorescence is often suppressed due to the absence of key functional groups or the presence of a quencher moiety in the precursor. After the Staudinger reaction, the fluorescence of the probe is restored either by recovering the functional group or by removing the quencher, enabling signal generation for detection. In 2008, Franzini et al. [41] reported a DNA detection method based on the combination of the Staudinger reduction and a 7‐hydroxycoumarin fluorophore. In this method, the target DNA was initially modified with an azide‐caged 7‐hydroxycoumarin, which is nonfluorescent. A complementary DNA strand bearing a phosphine group triggered the Staudinger reduction, uncaging the fluorophore in a sequence‐specific manner with DNA template. The fluorescence intensity generated by the released 7‐hydroxycoumarin correlated positively with the concentration of the target DNA, allowing its semi‐quantitative analysis. In the same year, Hangauer et al. [42]. reported a glycan labeling method that utilized azide sugars and a fluorescence resonance energy transfer (FRET)‐based phosphine probe for semi‐quantitative analysis in live cells (Figure 3a,b). The FRET‐based phosphine was initially quenched but became fluorescent upon reaction with azide‐modified sugars via nontraceless Staudinger ligation. Since azide sugars can be metabolically incorporated into cell‐surface glycans, this method enabled the fluorescent labeling of glycans on the cell membrane. The resulting fluorescence intensity was positively correlated with the abundance of labeled glycans, allowing for semi‐quantitative measurement in live‐cell conditions. Recently, Bajaj et al. [43] reported fluorescent glutamine and asparagine probes for detecting the distribution of colon cancer cells. In this method, glutamine and asparagine were conjugated to phosphines and then reacted with 4‐(azidomethyl)‐7‐hydroxycoumarin via a traceless Staudinger reaction to generate fluorescent probes. These probes were taken up and retained by the cells, and the resulting fluorescence intensity showed a positive correlation with the distribution and abundance of the cancer cells.

Besides fluorophores, other probe molecules have also been applied to enable semi‐quantification using Staudinger reactions. One example is luciferin, the substrate of the firefly bioluminescence reaction, which generates luminescence in the presence of luciferase. Cohen et al. [44] reported a real‐time imaging method for glycans on live cells using a nontraceless Staudinger reaction (Figure 3c). In this method, cell‐surface glycans were metabolically labeled with azide sugars, while luciferin was caged as the leaving group on a phosphine reagent **1**. Upon Staudinger ligation, luciferin was released and reacted with firefly luciferase expressed in a prostate cancer cell line, producing a luminescence signal. The photon flux was measured by a CCD camera and showed a positive correlation with glycan abundance. The authors reported that although the blank sample exhibited a low luminescence signal due to background luciferin release via ester hydrolysis, the method is significantly more sensitive than fluorescence‐based detection. Another example is the [^1^⁸F]‐labeled radioprobe, which is an essential molecule for PET, enabling noninvasive imaging of live cells and organs in vivo [45]. Gaeta et al. [46]. reported a radioprobe targeting the *γ*‐aminobutyric acid (GABA) receptor for PET imaging. The probe was synthesized via a traceless Staudinger ligation between a 4‐quinolone derivative with GABA receptor affinity and 2‐[^1^⁸F]fluoroethylazide. The detected radioactivity was positively correlated with the abundance of GABA receptors, allowing for semi‐quantitative mapping of GABA receptor distribution in the rat brain.

### 2.3 Application in Quantitative Analysis

**FIGURE 4:**
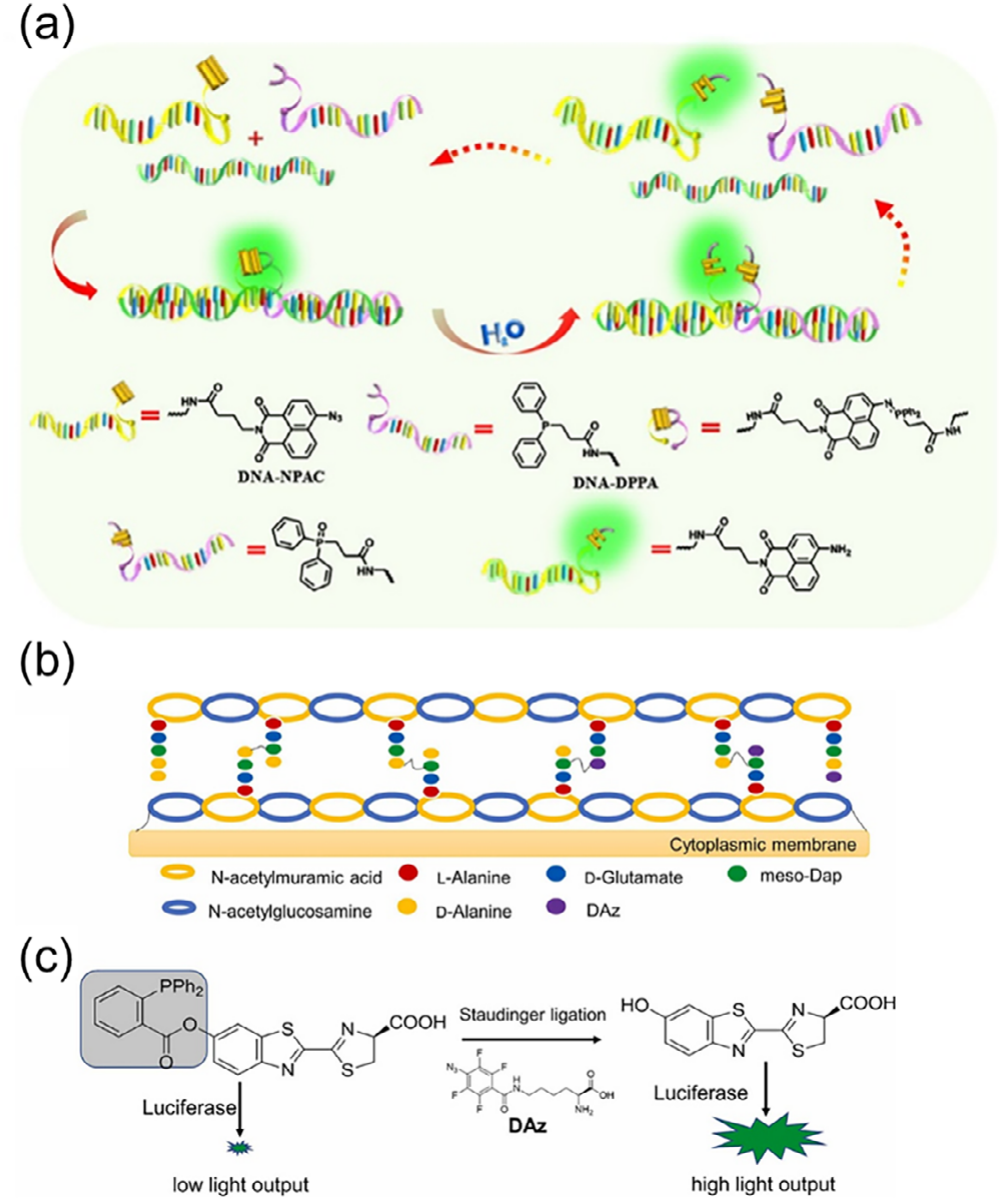
(a) Schematic illustration of HMSR‐probes. (b) Structure of peptidoglycan and (c) the reaction mechanism of TL‐PDC with Daz. Adapted from References [48] and [49] with permission. Copyright 2019, American Chemical Society and Copyright 2022 Elsevier.

Based on the semi‐quantitative analysis methodologies, recently the Staudinger ligation has been developed for application in quantitative biomolecular analysis. Notably, these applications often involve fluorophores or luminescent probes rather than biotin, suggesting that the complex detection protocols required for biotin‐based assays may reduce their accuracy when coupled with Staudinger ligation. For example, in 2019, Ma et al. [47] developed a fluorescent probe for quantifying nitroxyl, a key signaling molecule involved in various physiological processes. The probe, consisting of a naphthalene backbone and a 2‐(diphenylphosphino)benzoate moiety, releases the fluorophore via a nontraceless Staudinger ligation, resulting in a strong fluorescence signal. A calibration curve between fluorescence intensity and the concentration of Angeli's salt (a common nitroxyl source) was established, with a LOD of 43 nM. The probe demonstrated a broad working pH range (4–10), good selectivity in the presence of biologically relevant substances, and no cytotoxicity in HepG2 cells. In the same year, Xian et al. [48] reported a strategy using hybridization‐mediated Staudinger reduction probes (HMSR‐probes) for intracellular microRNA detection (Figure 4a). In this approach, phosphine‐ and azide‐modified DNA strands are brought into close proximity via DNA‐RNA hybridization of miRNA, triggering a Staudinger reaction that increase fluorescence intensity of product to 10 times larger than that of reactant. A linear relationship was observed between normalized fluorescence intensity and the logarithm of microRNA concentration. The method achieved a LOD of 1.3 × 10^−^^1^⁵ M in vitro, significantly lower than the 3.9 nM LOD for the CuAAC‐based system reported in the same condition. Although the CuAAC reaction exhibited a higher reaction rate, the authors attributed the lower LOD of the Staudinger ligation to its suitability for oligonucleotide template‐driven turnover amplification.

In 2022, Liang et al. [22] reported a method combining arylphosphine‐induced acceptor photoinduced electron transfer (a‐PET) and Staudinger ligation for highly specific and sensitive detection of glucose uptake in live cells. The probe, Glu‐HT‐Me—comprising an HT‐Me fluorophore and a phosphine moiety—was first introduced into the cytosol. Subsequently, azide‐modified glucose (AzGlu2) was taken up by the cells, where it underwent a traceless Staudinger ligation with Glu‐HT‐Me, triggering a fluorescence turn‐on response. A linear correlation between the fluorescence intensity at 560 nm and the concentration of AzGlu2 was established, with a reported LOD of 96 nM. In the same year, Song et al. [49]. reported a method combining metabolic labeling and the nontraceless Staudinger reaction to quantify and image peptidoglycan in bacterial cells (Figure 4b,c). Peptidoglycan was metabolically labeled using aryl azide‐modified D‐amino acids (DAz), while luciferin was caged as the leaving group in a phosphine compound. Upon undergoing the Staudinger reaction within genetically modified bacterial cells, luciferin was released and subsequently catalyzed by luciferase to generate a luminescence signal. The luminescence intensity correlated with the concentration of DAz, and a nonlinear fitting was used to establish this relationship. This method had also been applied to quantify the peptidoglycans with exogenously DAz‐labeled bacteria in mice.

## 3 Strain‐Promoted Azide‐Alkyne Cycloaddition (SPAAC)

### 3.1 Overview

Azide–alkyne cycloaddition, first introduced by Michael in the late 1890 and developed as a “click reaction” by Sharpless in 2001, is a fast, biocompatible, water‐ and air‐tolerant, and highly selective reaction between azide and alkyne groups [50]. The classic form of this reaction, known as copper(I)‐catalyzed azide–alkyne cycloaddition (CuAAC), uses a copper(I) catalyst to accelerate the reaction. However, concerns over copper‐induced cytotoxicity have limited its use in biological applications, particularly in in vivo systems. To overcome this limitation, strain‐promoted azide–alkyne cycloaddition (SPAAC) was developed as a copper‐free alternative. SPAAC exploits the ring strain of cyclooctyne derivatives (typically 7‐membered or larger rings) to drive the reaction forward without metal catalysts. Adronov and his coworkers [51] introduced the SPAAC in a previous review. Here, we briefly summarize its reaction mechanism, kinetics, and applications in biomolecules quantification.

**SCHEME 2:**
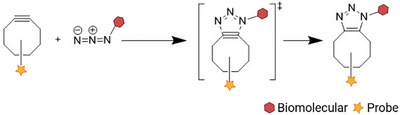
Concerted mechanism of the strain‐promoted alkyne‐azide cycloaddition. Adapted from Reference [52] with permission. Copyright 2017, Wiley.

The underlying mechanism of SPAAC (Scheme 2) is distinct from that of CuAAC, which requires a copper(I) catalyst to activate the alkyne and facilitate the formation of cyclic intermediates [52, 53]. In contrast, SPAAC proceeds without a metal catalyst. The reaction is initiated by the inherent ring strain in cyclic alkynes—typically those with eight or more carbon atoms—which destabilizes the carbon–carbon triple bond and makes it more reactive [54]. This effect was first observed by Wittig and Krebs in 1961, when they reported a highly exothermic reaction between cyclooctyne and phenyl azide, describing it as an “explosion reaction” [55]. After initiation, the strained alkyne undergoes a [3+2] dipolar cycloaddition with the azide to form a stable 1,2,3‐triazole ring through a cyclic transition state.

From a kinetic perspective, SPAAC follows second‐order kinetics with reported rate constants ranging from 0.01 to 60 M^−^^1^ s^−^^1^, corresponding to an estimated half‐life of about 0.5 h when both reactants are present at 10 µM and the rate constant is 60 M^−^^1^ s^−^^1^, which is comparable to the lifetimes of short‐lived regulatory proteins that typically range from minutes to hours [12, 56, 57]. This relatively fast reaction rate is primarily attributed to the ring strain in cycloalkynes, which destabilizes the carbon–carbon triple bond and drives the reaction forward spontaneously [54]. The reaction rate is also influenced by the structure of the azide and various reaction conditions. For example, aryl azides—particularly those bearing electron‐withdrawing groups—tend to decrease the reaction rate [58]. In contrast, electron‐rich cyclooctynes can significantly enhance reactivity when paired with such azides. Solvent effects are also notable; aqueous solvents are generally preferred due to their ability to improve solubility and reaction efficiency. Additionally, the reaction performs optimally at pH 7 or higher, although stability concerns may arise at pH values above 8 [59].

SPAAC, as a copper‐free click reaction, eliminates the cytotoxicity concerns associated with CuAAC while retaining the desirable attributes of click chemistry. It exhibits high selectivity toward azide groups, even in vivo, and combines excellent biocompatibility with rapid reaction kinetics [60]. The azide group can be introduced into various biomolecules with stability under various conditions as mentioned in the Staudinger reaction. Strained cyclic alkynes such as Dibenzocyclooctyne (DBCO) and bicyclo[6.1.0]non‐4‐yne (BCN) are commonly conjugated with probe molecules and exhibit stability comparable to azides, but they are more sensitive to reducing agents—BCN is unstable in the presence of glutathione (GSH), while DBCO is degraded by both TCEP and GSH [36]. These features make SPAAC a versatile and powerful tool for both in vitro and in vivo applications. As a result, it has been widely adopted for semi‐quantitative and quantitative analyses in biological systems.

### 3.2 Application in Semi‐Quantitative Analysis

**FIGURE 5:**
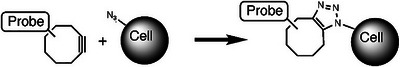
Schematic illustration of SPAAC reaction in labeling cells. Reprint from Reference [54] with permission. Copyright 2004, American Chemical Society.

When used for semi‐quantitative analysis, the two substrates in the SPAAC reaction—azides and cycloalkynes—lack intrinsic detectable signals. Therefore, they are typically conjugated with probe molecules to enable detection. Biotin is one of the most commonly used probes, often combined with fluorescent dyes or Western blotting, as described previously in the context of Staudinger ligation. This biotin‐based detection strategy is also widely applied in SPAAC reactions. In 2004, Agard et al. [54] reported a method that replaced Staudinger ligation with SPAAC for labeling biomolecules in living cells (Figure 5). In their study, sialic acid residues on the glycan surface were metabolically labeled using an azide‐functionalized sugar, peracetylated N‐α‐azidoacetylmannosamine (Ac_4_ManNAz). The complementary cyclooctyne was conjugated with the biotin probe. After SPAAC labeling, biotin‐tagged sialic acids were detected and quantified by Western blotting using HRP–anti‐biotin and by flow cytometry using FITC–avidin staining. Notably, this method demonstrated no cytotoxicity toward cells, addressing the limitations of Staudinger ligation. Similarly, in 2011, Mbua et al. [61] developed a related approach to assess glycosylation defects via metabolic labeling and SPAAC. In their work, Ac_4_ManNAz was again used to introduce azide groups into sialic acids, but the biotinylated cyclooctyne was replaced by 4‐dibenzocyclooctynol (DIBO). Following SPAAC conjugation, the labeled sialic acids were stained with avidin–AlexaFluor488, and fluorescence intensity was measured. Confocal microscopy enabled visualization and semi‐quantitative analysis of sialic acid abundance on the cell surface. This method showed improved labeling efficiency, with a reported second‐order rate constant of 0.2590 M^−^^1^ s^−^^1^. This biotin‐conjugated SPAAC strategy has also been applied to the semi‐quantitative analysis of other biomolecules. Sabale et al. [39] reported a method utilizing this approach to label PNAs, which were then used for the semi‐quantification of DNA or RNA within cells. Similarly, Marchand et al. [62] employed this technique to label recombinant adeno‐associated viruses (rAAVs), enabling the semi‐quantitative analysis of viral proteins.

SPAAC reactions can also be directly coupled with fluorophores for the semi‐quantitative analysis of biomolecules based on their fluorescence properties. These fluorophores can be conjugated to either azide‐functionalized compounds or cycloalkynes. In 2011, Marks et al. [63] reported a method utilizing SPAAC to label DNA for semi‐quantitative analysis via fluorescence intensity. In their study, DIBO was modified onto peptides and shown to be stable under various conditions, including standard polymerase chain reaction (PCR), which enabled the preparation of DIBO‐modified DNA. This DNA was subsequently labeled with azide‐functionalized fluorophores through SPAAC chemistry, and the resulting fluorescence—using Texas Red and SYBR Green I dyes—correlated positively with the amount of labeled DNA. In 2015, Tian et al. [64] developed a SPAAC‐based approach using Alexa Fluor 488 to semi‐quantitatively label and image proteins on lipid membranes. In this method, Alexa Fluor 488 was conjugated to a cyclooctyne (DIBO), while the azide group was genetically encoded into G protein‐coupled receptors (GPCRs) by incorporating p‐azido‐phenylalanine (azF). After SPAAC labeling, the distribution of GPCRs on lipid bilayers was visualized and semi‐quantified via fluorescence intensity. In the same year, Zayas et al. [65] extended SPAAC chemistry to nucleosides, nucleotides, and living cancer cells for direct fluorescent imaging. A series of azide‐functionalized nucleosides were reacted with DIBO or other cyclooctynes, yielding triazole products with inherent fluorescent properties. This enabled direct fluorescence imaging of cancer cells without the need for additional dyes.

SPAAC chemistry has also been widely applied in radiochemistry to enable semi‐quantitative analysis of biomolecules via PET imaging. In such applications, the radiolabeled probe can be introduced either through an azide or a cycloalkyne component. In 2011, Campbell–Verduyn et al. [66] developed a method using the SPAAC reaction to label bombesin with fluorine‐^18^F azide for cancer cell imaging. Bombesin, a peptide with high affinity for certain cancer cells, was functionalized with a cycloalkyne derivative, aza‐dibenzocyclooctyne (aza‐DBCO), while the radioactive fluorine was introduced through an ^18^F‐labeled azide. Through SPAAC conjugation, bombesin was efficiently radiolabeled, allowing its distribution in biological systems to be visualized and semi‐quantified using PET analysis. In 2013, Hausner et al. [21] reported a complementary strategy in which the cycloalkyne was radiolabeled. In this method, a DIBO derivative labeled with ^18^F, [^18^F]FBA‐C6‐ADIBO, was used as the SPAAC probe. The azide‐functionalized PEGylated A20FMDV2 peptide, which selectively targets the integrin *α*_v_ *β*_6_—a surface receptor linked to the prognosis of several cancers—was conjugated with the radiolabeled DIBO via SPAAC reaction. The resulting structure, [^18^F]FBA‐C6‐ADIBO‐N_3_‐PEG_7_‐A20FMDV2, enabled PET‐based imaging and semi‐quantification of integrin *α*_v_ *β*_6_‐expressing cancer cells. Beyond fluorine‐based radioprobes, other radioisotopes have also been employed via SPAAC reaction. Zeng et al. [67] reported a ^64^Cu‐labeled probe synthesized via SPAAC chemistry for the semi‐quantitative imaging of epidermal growth factor receptor (EGFR) expression in tumors using PET analysis. Similarly, Jadhav et al. [68] developed a ^68^Ga‐labeled probe to visualize infarcted regions in mice. The probe precursor was synthesized through a SPAAC reaction between a cyclooctyne‐functionalized oligonucleotide and 3‐azidopropyl hexasaccharide, followed by radiolabeling with ^68^Ga. These examples demonstrate that SPAAC chemistry is a powerful tool in radiochemical semi‐quantification due to its excellent biocompatibility and rapid kinetics.

### 3.3 Application in Quantitative Analysis

**FIGURE 6:**
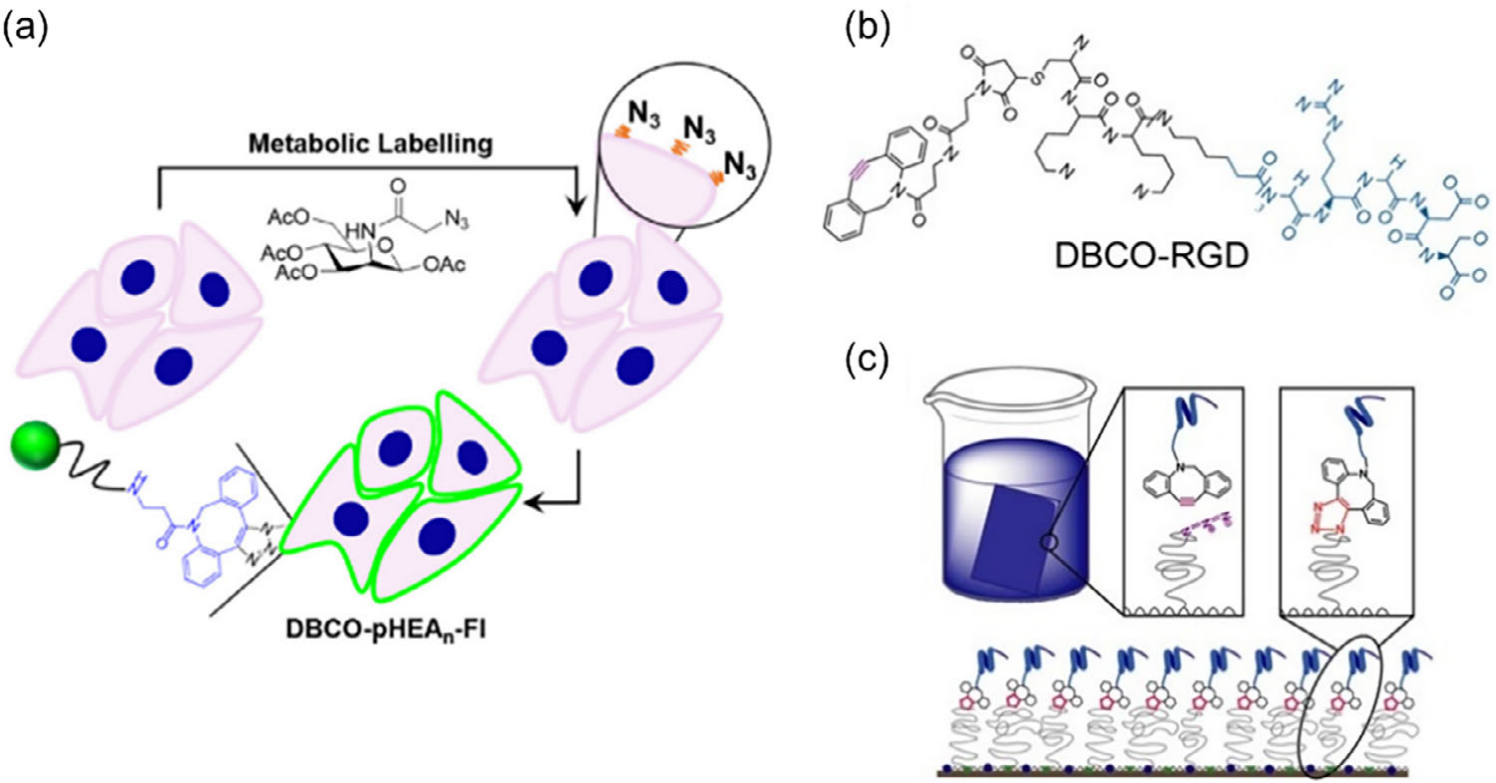
(a) Schematic illustration of Metabolic labeling and SPAAC fluorescence coating. (b) Structure of DBCO‐RGD and (c) Illustration of SPAAC‐based immobilization of DBCO‐RGD. Reprint from References [69] and [72] with permission. Copyright 2019, American Chemical Society and Copyright 2018, Elsevier under CC‐BY 4.0 license.

SPAAC chemistry, owing to its excellent biocompatibility and rapid kinetics, has also been applied to the quantitative analysis of biomolecules combined with fluorescence spectroscopy and luminesce spectroscopy. Tomás et al. [69] developed a method combining SPAAC reaction and fluorescence‐based techniques to quantify membrane polymer coatings and assess their stability (Figure 6a). In this approach, glycans in the cell membrane were metabolically labeled with azide sugars, and poly(hydroxysthyl acrylamide) (pHEA) polymers modified with DBCO and fluorophores (DBCO‐pHEA‐Fl) were grafted onto the surface via SPAAC reaction. The amount of grafted polymer correlated positively with DBCO concentration and was quantified by flow cytometry and confocal microscopy based on fluorescence intensity. A standard calibration curve revealed a linear relationship between DBCO‐pHEA‐Fl concentration and relative fluorescence intensity in the range of 1.25–10 mg·mL^−^^1^. Mao et al. [70] reported a time‐resolved chemiluminescence immunoassay (TRCLIA) combined with SPAAC labeling for the detection of tumor markers. Carcinoembryonic antigen (CEA) and neuron‐specific enolase (NSE) in human serum were immobilized on carboxylate‐modified polystyrene microspheres (CPSMS), while their corresponding antibodies were labeled with chemiluminescent probes—horseradish peroxidase (HRP) and alkaline phosphatase (ALP)—via SPAAC reaction. The signal of CEA and NSE was recorded at 0.5 s and 20 min. The luminescence intensity showed a strong linear correlation with CEA and NSE concentrations over the ranges of 0.1–64 ng·mL^−^^1^ and 0.05–64 ng·mL^−^^1^, respectively. The LODs were 0.085 ng·mL^−^^1^ for CEA and 0.044 ng·mL^−^^1^ for NSE.

Mass spectrometry (MS) has also been integrated with SPAAC chemistry to support the quantitative analysis of biomolecules. Although MS lacks spatial resolution, it provides superior sensitivity for quantification. Geissinger et al. [71] utilized SPAAC to label peptides pAzF‐SC‐2‐16 and BDP‐SC‐2‐16 in live cells for analysis by LC‐MS. The probe BDP‐FL‐PEG_4_‐DBCO, bearing a cycloalkyne group, enabled both semi‐quantitative fluorescence detection via confocal microscopy and quantitative MS analysis. Following SPAAC conjugation, fluorescence intensity correlated with peptide abundance, and MS peak area showed a linear relationship with peptide concentration. The LOD for BDP‐SC‐2‐16 was reported as 0.0002 amol per cell. Mertgen et al. [72] further demonstrated a SPAAC‐based strategy to graft bioactive molecules onto a PAcrAm graft copolymer and quantify them using Time‐of‐Flight Secondary Ion Mass Spectrometry (ToF‐SIMS) (Figure 6b,c). The polymer was azide‐functionalized, while active molecules such as RGD peptide and biotin were DBCO‐modified. Following SPAAC conjugation, ToF‐SIMS analysis revealed a positive correlation between signal intensity and the surface concentration of DBCO‐RGD and DBCO‐Biotin, with LODs of 0.15 µM and 0.61 µM, respectively—over 40‐fold lower than those obtained via spectroscopic ellipsometry. Notably, the biotin‐labeling strategy also holds potential for accurate, rather than solely semi‐quantitative analysis. Recently, Hou et al. [73]reported a method combining SPAAC chemistry with MS to quantify Bruton's tyrosine kinase (BTK) in live cells. An azide‐modified ibrutinib probe (Ibt‐N_3_) was designed to permeate cells and selectively bind BTK. Following this, DBCO‐DOTA‐Eu was conjugated to the probe via SPAAC reaction. Although a standard calibration curve was not presented, the method enabled absolute quantification of intracellular BTK, reported as 61.28 ng per 10⁶ cells.

**FIGURE 7:**
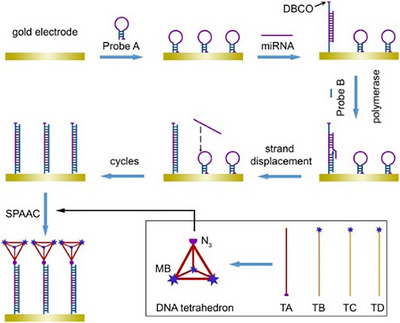
Illustrations of the Tetrahedral Nanotags Assembled from Four Single‐Stranded DNA and the Electrochemical Sensing Strategy Based on Strand Displacement Amplification and SPAAC Ligation. Reprinted from Reference [23] with permission. Copyright 2024, American Chemical Society under CC‐BY‐NC‐ND 4.0.

Beyond traditional analytical techniques, electrochemical methods have also been combined with SPAAC reaction due to their high sensitivity, rapid response, and real‐time analysis capabilities [74]. Electrochemical detection relies on current or voltage signals generated through redox reactions, requiring substrates with conductive or redox‐active properties. Ma et al. [23] developed a novel electrochemical strategy to quantify miRNA biomarkers using DNA tetrahedrons as signal reporters. In this system, target miRNA triggers the structural opening of hairpin Probe A, exposing a DBCO group that reacts with azide‐labeled DNA tetrahedrons via SPAAC reaction (Figure 7). This selective reaction immobilizes signal probes on the electrode surface. Three methylene blue (MB) molecules at the vertices of each DNA tetrahedron serve as redox‐active centers, repeatedly oxidized and reduced in the presence of TCEP, generating amplified current signals. The peak current exhibited a linear correlation with the logarithm of miRNA concentration across the range of 10^−^^1^⁶ to 10^−^^1^ ^1^ M, with a calculated LOD of 75 aM.

## 4 Inverse Electron‐Demand Diels–Alder Reactions (IEDDA)

### 4.1 Overview

The IEDDA reaction was first introduced by Blackman in 2008 and is currently regarded as the fastest bioorthogonal reaction known [75]. It is derived from the classical Diels–Alder (DA) reaction but involves different electronic requirements: an electron‐poor diene, typically 1,2,4,5‐tetrazine (Tz), and an electron‐rich dienophile such as trans‐cyclooctene (TCO). Due to its exceptionally rapid kinetics, high selectivity, and excellent biocompatibility, the IEDDA reaction has become a powerful tool for both in vitro and in vivo applications. de la Torre and his coworkers [76] introduced the IEDDA reaction in a previous review. Here, we briefly summarize its reaction mechanism, kinetics, and applications in biomolecules quantification.

**SCHEME 3:**
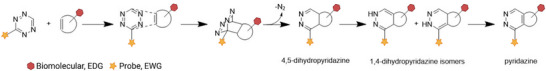
Schematic representation of the reaction mechanism between a dienophile and a tetrazine. Adapted from Reference [18] with permission. Copyright 2024, RSC under CC‐BY 3.0.

Mechanistically, the IEDDA reaction proceeds via a [4+2] cycloaddition between the electron‐deficient diene and the electron‐rich dienophile, resulting in the formation of a six‐membered ring (Scheme 3) [18]. This inverse electron‐demand differs from the classical Diels–Alder reaction, which typically involves an electron‐rich diene and an electron‐poor dienophile. In the IEDDA reaction, the ─C═N─N═C─ moiety of the tetrazine reacts with the alkene in a 1,4‐addition, forming a highly strained bicyclic intermediate. This intermediate rapidly decomposes with the release of one molecule of nitrogen gas (N_2_), yielding a 4,5‐dihydropyridazine via a retro‐Diels–Alder process. The resulting 4,5‐dihydropyridazine can then either undergo oxidation to form a stable pyridazine product or isomerize to its corresponding 1,4‐dihydro isomers.

The IEDDA reaction, known as one of the fastest bioorthogonal reactions, exhibits a second‐order rate constant ranging from 1 to 10⁶ M^−^^1^ s^−^^1^ and an estimated half‐life as short as 0.1 s when both reactants are present at an initial concentration of 10 µM and the rate constant is 10⁶ M^−^^1^ s^−^^1^ [12, 77]. Such rapid kinetics and ultra‐short half‐lives indicate that quantification or signal generation process can occur within a very short time frame, minimizing interference from complex biological environments and enabling real‐time analysis with high temporal resolution [78]. The origin of this rapid kinetics can be rationalized using frontier molecular orbital (FMO) theory, which explains how the reactivity is influenced by the energy gap between the HOMO of the dienophile and the LUMO of the diene [18]. This energy gap can be significantly reduced by introducing electron‐withdrawing groups (EWGs) on the diene or electron‐donating groups (EDGs) on the dienophile, thereby enhancing the reaction rate [79, 80]. Additionally, increasing the ring strain in the dienophile raises its HOMO energy and boosts its reactivity [81]. Stereochemistry and steric effects also play important roles—certain stereoisomers are more reactive, and bulky substituents can hinder the reaction due to steric congestion [77, 82]. Beyond the molecular structure, solvent effects are notable: the IEDDA reaction is typically faster in protic solvents than in water [83]. The effect of pH is relatively minor, with slightly faster rates observed under mildly acidic conditions [84]. The IEDDA reaction is highly selective between tetrazines and strained alkenes/alkynes, reflecting its strong bioorthogonality; however, it exhibits limited stereoselectivity, resulting in both endo‐ and exo‐addition products [85].

Besides its fast kinetics, excellent biocompatibility, and bioorthogonality, the IEDDA reaction also offers highly detectable substrates for analytical applications. The unique conjugated structure of the diene, Tz, enables fluorescence “turn‐on” mechanisms via IEDDA reaction, particularly when appropriate substituents are introduced [18]. This feature significantly reduces background signals, enhancing the reliability and sensitivity of detection. Moreover, the diene and dienophile can be readily conjugated to various biomolecules, especially proteins which could label via genetic code expansion. These advantageous properties make IEDDA an ideal tool for both semi‐quantitative and quantitative analysis of biomolecules, and its use has become widespread in various bioanalytical contexts [86]. However, probe stability must be carefully considered when applied the IEDDA reaction, as the high reactivity of tetrazines can also lead to degradation under certain conditions. For example, highly reactive tetrazines can decompose at pH 8.0 or in the presence of redox agents such as TCEP and dehydroascorbic acid (DHA), or in CuAAC buffers, whereas less reactive methyl‐substituted tetrazines remain stable under pH 8.0 or DHA condition [36].

### 4.2 Application in Semi‐Quantitative Analysis

**FIGURE 8:**
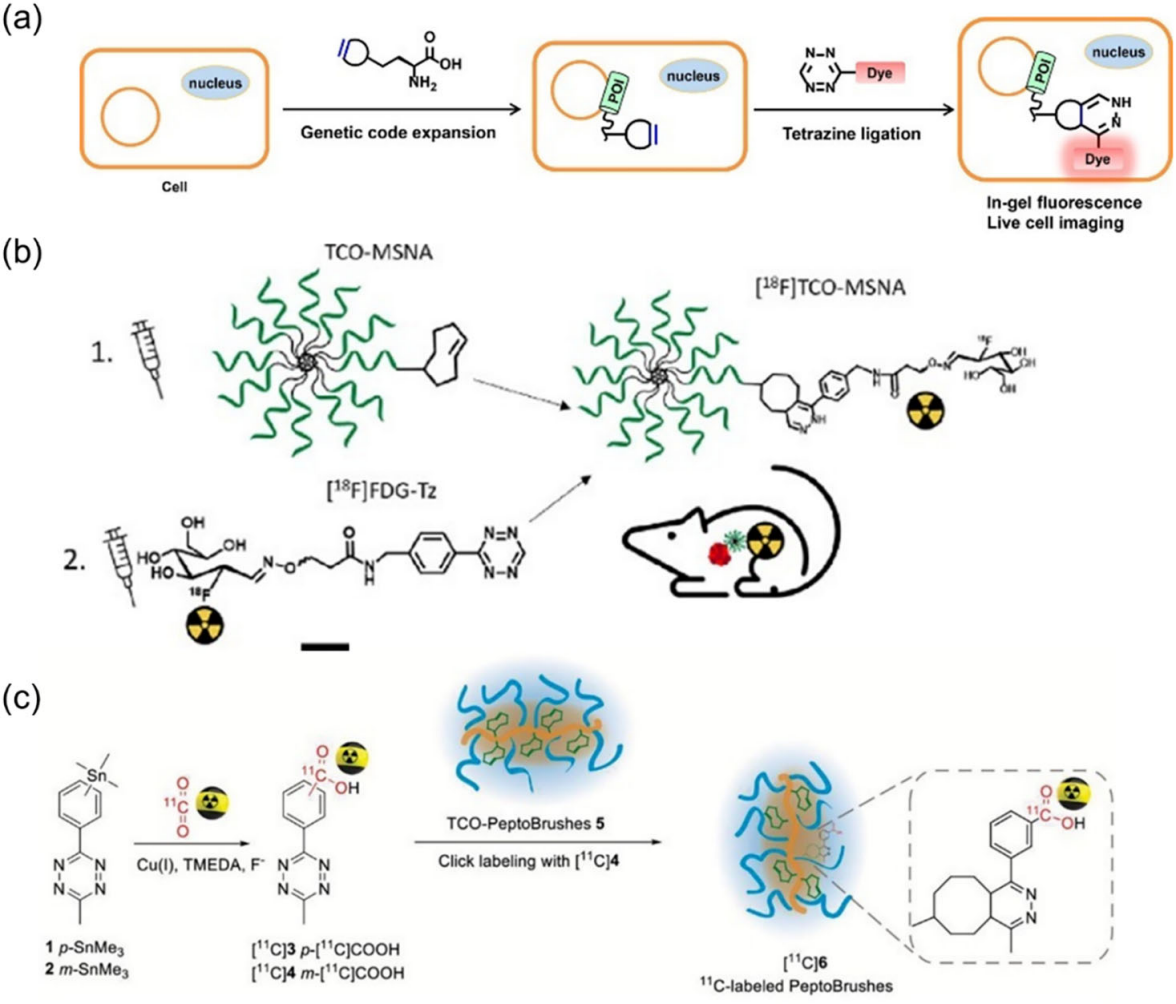
(a) Scheme for site‐specific fluorescence labeling and imaging of intracellular proteins of interest (POI) in live cells using unnatural amino acid incorporation via genetic code expansion and bioorthogonal tetrazine ligation reaction. (b) Schematic illustration of Tetrazine Glycoconjugate for Pretargeted PET Imaging of trans‐Cyclooctene‐Functionalized Molecular Spherical Nucleic Acids. (c) General strategy for application of [^11^C]carboxylated tetrazines to ^11^C‐labeling of TCO‐containing PeptoBrushes. Adapted from References [88], [96], and [97] with permission. Copyright 2016, American Chemical Society, Copyright 2023, American Chemical Society under CC‐BY 4.0 and Copyright 2021, Wiley under CC‐BY 4.0.

The IEDDA reaction has been widely applied in the semi‐quantitative analysis of biomolecules, particularly using fluorescence‐based techniques. The unique conjugated structure of the diene, Tz, enables fluorescence turn‐on strategies during the IEDDA reaction. A common approach involves modifying Tz with fluorophores such as coumarin, BODIPY FL, or xanthene. These fluorophores are initially quenched due to energy transfer or absorption by Tz, which absorbs light in the 500–530 nm range. Upon undergoing the IEDDA reaction, the conjugated structure of Tz is disrupted, restoring the fluorescence signal. Carlson et al. [87] developed a method utilizing the IEDDA reaction to label antibodies and visualize their distribution in cells. TCO was conjugated to lysine residues of the antibody via amine‐reactive chemistry, while Tz was linked to a BODIPY fluorophore. Initially quenched by Tz, the fluorescence signal increased by several hundred‐fold after the IEDDA reaction. The fluorescence intensity correlated with the abundance of the antibody, and its spatial distribution in cells was visualized using confocal microscopy. Similarly, Peng et al. [88]. reported a strategy combining IEDDA chemistry and Tz‐BODIPY derivatives to semi‐quantify IFITM3, a small membrane‐associated protein (Figure 8a). In this approach, TCO was site‐specifically incorporated into the protein via genetic code expansion. The abundance and localization of IFITM3 were semi‐quantified through fluorescence intensity captured by confocal microscopy. Fluorophores such as coumarin and xanthene have been used in IEDDA‐based fluorescence turn‐on strategies. Memetics et al. [89]. developed ultrafluorogenic coumarin–tetrazine probes for imaging antibody‐labeled proteins in live cells, showing fluorescence recovery upon IEDDA reaction. Similarly, Wu et al. [90] applied xanthene–tetrazine probes to visualize antibody distribution, both demonstrating fluorescence intensity correlated with target abundance. Wang et al. [91] further extended this strategy to monitor nutrient uptake in live cells. They designed a dually quenched CFSE–tetrazine probe that becomes fluorogenic upon esterase activation in the cytosol and becomes fluorescent only after reacting with strained alkenes via IEDDA. This approach enabled real‐time tracking of the uptake of fatty acids, sugars, and amino acids in primary immune cells based on fluorescence intensity.

Another approach to achieve fluorescence turn‐on for semi‐quantification is to use nonfluorescent tetrazine and alkene derivatives that form a fluorescent product after the IEDDA reaction. Shang et al. [92]. reported a fluorogenic bioorthogonal reaction using tetrazine and genetically encoded styrene‐tagged proteins. The resulting product, 4‐phenyl‐3,6‐di(pyridin‐2‐yl)‐1,4‐dihydropyridazine (PDHP), exhibited strong fluorescence and good stability in various solvents and physiological pH. Protein labeling was confirmed via western blotting, and fluorescence intensity measured by confocal microscopy correlated with protein distribution in vivo. Vázquez et al. [93] reported a method using specific TCO isomers and Tz to generate fluorescent 1,4‐dihydropyridazine products via IEDDA for peptide labeling in cells. Tz was conjugated to a model peptide via an N‐terminal lysine, and fluorescence was triggered upon reaction with TCO. They also tested the reverse approach—modifying the peptide with TCO and reacting with Tz—but found that certain TCO substitutions abolished fluorescence, emphasizing the need for careful probe design. Fluorescence intensity, measured by confocal microscopy, correlated with peptide distribution in cells.

Another widely used technique with the IEDDA reaction is PET imaging, which enables semi quantitative analysis of biomolecule distribution using radiolabeled probes. Because these probes have a short half‐life, the method requires a rapid and selective labeling process, which the IEDDA reaction can efficiently provide. [^18^F] is the most common radio probe. Meyer et al. [94] reported a method utilizing the IEDDA reaction for ^18^F pretargeted PET imaging of cancer cells. In this approach, the 5B1 antibody targeting CA19.9‐expressing BxPC3 pancreatic cancer xenografts was modified with TCO via incubation with the activated succinimidyl ester of TCO. Separately, the [^1^⁸F] radioprobe was chemically linked to tetrazine through AlCl_3_‐catalyzed labeling. Following the IEDDA reaction, the target protein on cancer cells was radiolabeled, and its distribution was visualized using PET imaging. Pieve et al. [95] also developed a method combining the IEDDA reaction with PET imaging to monitor human epidermal growth factor receptor 3 (HER3), a biomarker associated with certain cancers. Similar to the previously described approach, their method used TCO, but in this case, it was maleimide‐functionalized and conjugated to HER3 via cysteine residues. More recently, Auchynnikava et al. [96] reported a method to image nucleic acids using PET imaging and the IEDDA reaction (Figure 8b). In this approach, TCO was attached to molecular spherical nucleic acids (MSNAs) containing an anti‐HER2 oligonucleotide sequence related to cancer. The tetrazine was radiolabeled with [^1^⁸F]FDG, synthesized via nucleophilic [^1^⁸F]fluorination from tetra‐acetylated mannose triflate. Through the IEDDA reaction, the target MSNA was radiolabeled, and its distribution was visualized using PET imaging.

Besides [^1^⁸F], other radionuclides such as [^1^ ^1^C], [⁶⁴Cu], [⁶⁸Ga], and [⁸⁹Zr] have also been incorporated into biomolecules via the IEDDA reaction. García–Vázquez et al. [97] reported a novel [^1^ ^1^C]‐labeled tetrazine probe synthesized through a carboxylation reaction, which was used to monitor the fate of macromolecules like biopolymers using PET imaging (Figure 8c). In their method, the macromolecule PeptoBrushes was first modified with TCO via a condensation reaction between amine and carboxylic acid groups. The TCO‐modified PeptoBrushes then underwent the IEDDA reaction with the [^1^ ^1^C]‐labeled tetrazine to yield radiolabeled PeptoBrushes. The distribution and metabolic fate of these labeled macromolecules were subsequently visualized using PET imaging. Shi et al. [98]. reported a method using a [⁶⁴Cu]‐labeled probe and the IEDDA reaction to monitor colorectal tumors expressing vascular endothelial growth factor (VEGF). The modification strategy followed a reversed approach: the strained alkene was labeled with [⁶⁴Cu] by reacting Reppe anhydride derivatives with p‐SCN‐Bn‐NOTA, forming [⁶⁴Cu]‐NOTA‐TD. Meanwhile, the tetrazine was conjugated to bevacizumab, an antibody with high affinity for VEGF‐expressing cells. The subsequent IEDDA reaction enabled radiolabeling of the antibody, which was then tracked via PET imaging. Lambidis et al. [99] described the synthesis of two [⁶⁸Ga]‐labeled tetrazines, which were used to radiolabel two different TCO‐functionalized nanoparticles through the IEDDA reaction, allowing their distribution to be visualized by PET imaging. Lumen et al. [100] also reported a strategy for tumor imaging by performing the IEDDA reaction between a [⁸⁹Zr]‐labeled tetrazine and the TCO‐functionalized antibody U36, enabling PET‐based visualization of tumor localization.

In radiochemistry, another luminescence imaging method called Cerenkov luminescence imaging (CLI) has also been applied with the IEDDA reaction to semi‐quantify the distribution of radiolabeled biomolecules. CLI captures photons emitted from radioactive probes through Cerenkov radiation and correlates the abundance of the probe with the luminescence intensity. This method offers advantages such as low cost, broad radionuclide compatibility, and simple equipment setup for real‐time analysis [101]. D'Onofrio et al. [102] reported a method using the IEDDA reaction to pretarget radiocomplexes toward tumor cells. In this approach, two types of radiocomplexes, [⁹⁰Y] and [^1^ ^1^ ^1^In], were modified with Tz and DOTA‐based chelators. The bombesin antagonist PEG_4_‐AR, which has affinity for tumor cells, was modified with TCO. After the IEDDA reaction between Tz and TCO, the resulting radiocomplex exhibited tumor‐specific binding. CLI was then applied to semi‐quantify the accumulation of the radiocomplex based on luminescence intensity. Mack et al. [103] also reported a pretargeting approach using the IEDDA reaction to treat serous ovarian cancer. In this method, the humanized anti‐MUC16 monoclonal antibody AR9.6 was modified with TCO to provide tumor‐targeting ability. The cytotoxic component [^2^ ^2^⁵Ac]Ac‐mcp‐PEG_8_ was modified with Tz. After the IEDDA reaction, the [^2^ ^2^⁵Ac]‐labeled compound specifically accumulated in tumor tissue. CLI was applied to confirm the accumulation of the labeled compound by measuring luminescence intensity.

### 4.3 Application in Quantitative Analysis

**FIGURE 9:**
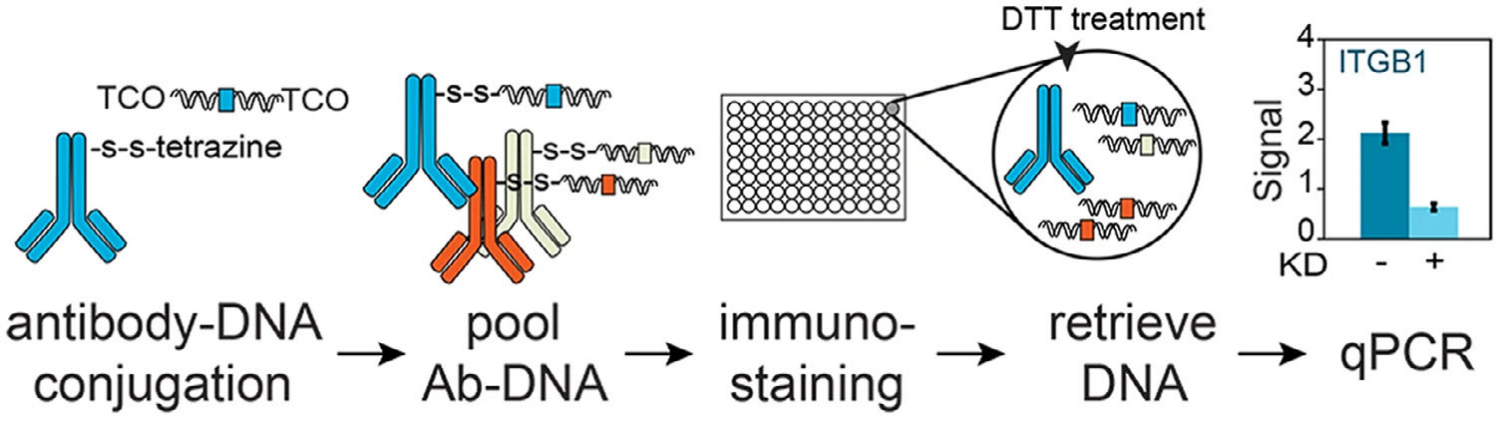
Schematic illustration of Immuno‐PCR method using antibody‐dsDNA conjugates. Reprinted from Reference [104] with permission. Copyright 2016, The Author(s) under CC‐BY 4.0.

The IEDDA reaction, known for its fast kinetics, high selectivity, and good biocompatibility, offers a powerful tool for the quantitative analysis of biomolecules. However, reported applications in this area remain surprisingly rare. A possible reason is that the IEDDA reaction is typically applied to large biomolecules, which are inherently more difficult to quantify. One notable example of its use in quantitative analysis is its integration with Immuno‐PCR. van van Buggenum et al. [104] reported a cleavable antibody–DNA conjugate prepared via the IEDDA reaction, which enables the release of double‐stranded DNA (dsDNA) for quantification by Immuno‐PCR (Figure 9). In this method, antibodies targeting markers such as ITGB1 and the differentiation marker transglutaminase 1 (TGM1) were functionalized with Tz using NHS chemistry. dsDNA was enzymatically modified with N_3_‐dATP, followed by a SPAAC reaction with DBCO‐TCO to introduce TCO functionality. The final antibody–DNA conjugates were generated via the IEDDA reaction, and the cleavable dsDNA was subsequently quantified by Immuno‐PCR. The study demonstrated a linear correlation between antibody concentration and Immuno‐PCR signal, with a better correlation coefficient (R^2^) than the standard in‐cell western (ICW) method. The LODs in this Immuno‐PCR were 0.095 and 0.094 for ITGB1 and TGM1, respectively, which were significantly lower than the 0.358 and 0.353 values of ICW.

Another application of the IEDDA reaction in quantitative analysis is its integration with fluorescence ratiometric techniques. Feng et al. [105] developed a ratiometric fluorescence probe based on the IEDDA reaction for ATP quantification in vitro and in living cells. The probe (RT‐1) was formed by reacting Tz‐conjugated rhodamine (ATP‐responsive, 584 nm) with TCO‐NHS, yielding an additional intrinsic fluorescence at 485 nm. The fluorescence ratio (I_584_/I_485_) showed a linear response to ATP across 0–2.5 mM and 2.0–10.0 mM, with LODs of 0.0354 mM and 0.0121 mM, respectively.

## 5 2‐Cyanobenzothiazole Cysteine Condensation (CBT‐Cys)

### 5.1 Overview

The 2‐cyanobenzothiazole (CBT)–cysteine (Cys) condensation reaction was first reported as a bioorthogonal reaction by Liang et al. in 2010, inspired by the natural biosynthesis of luciferin in fireflies [106]. In the biological context, CBT reacts with Cys to form luciferin, which then produces a luminescent signal in the presence of Mg^2^⁺, ATP, and luciferase [107]. Owing to its biological origin, this reaction demonstrates excellent biocompatibility and biosafety, making it suitable for applications in live‐cell systems [108]. Liang and his coworkers [109] introduced the CBT‐Cys condensation reaction in a previous review. Here, we briefly summarize its reaction mechanism, kinetics, and applications in biomolecules quantification.

**SCHEME 4:**
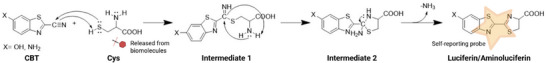
CBT‐Cys condensation reaction mechanism. Adapted from Reference [109] with permission. Copyright 2023, The Author(s) under CC‐BY 4.0.

Mechanistically, the CBT moiety reacts with a 1,2‐aminothiol group, typically located at the N‐terminus of a Cys residue, to form a stable cyclic product (Scheme 4) [109]. The reaction begins with a nucleophilic attack by the thiol group on the cyano carbon of CBT, accompanied by proton transfer to generate intermediate 1 featuring an imine unit. Subsequently, the amino group of Cys attacks the imine carbon and a proton transfer to the imine unit, leading to the formation of intermediate 2. A final proton transfer and elimination of an amino group yield the luciferin or aminoluciferin product, along with the release of a small molecule ammonia.

Kinetically, this condensation follows a second‐order rate law, with a reported rate constant of 9.19 M^−^^1^·s^−^^1^ under physiological conditions, giving an estimated half‐life of ∼3 h at 10 µM for both reactants and ∼13 min with a 10‐fold excess of one reactant [12, 110]. These values exceed those of standard azide–alkyne click reactions, indicating the CBT–Cys reaction's rapid, click‐like behavior and its potential applicability to diverse biomolecular targets [111]. Notably, the reaction is pH‐sensitive: a significant decrease in reaction rate is observed when the pH drops from 7.4 to 5.0 [112]. Conversely, higher pH values (>8.0) are also unfavorable, as CBT becomes prone to hydrolysis under alkaline conditions [113]. This narrow optimal pH range highlights the importance of carefully controlled conditions in biological applications. CBT–Cys condensation is selective for a free amino and thiol group, a characteristic feature of N‐terminal cysteine [114]. Its applicability can be limited by other thiols in the system or structural constraints on the target protein [108]. C‐terminal cysteine labeling could potentially be achieved using alternative bioorthogonal functional groups, such as hydrazine‐to‐azide modification [115].

Due to its mild reaction conditions, high chemoselectivity, and excellent biological compatibility, the CBT–Cys condensation has emerged as a valuable tool in bioorthogonal chemistry. D‐Cys can serve as a stable probe within cells, as supported by the natural intracellular presence of L‐Cys; however, endogenous cysteine may interfere with detection by competing with the target analyte and the cellular metabolism of D‐Cys should also be considered [116]. In addition, Its products—luciferin or aminoluciferin derivatives—exhibit unique fluorescent and bioluminescent characteristics [117]. Luciferin, for instance, shows fluorescence with excitation at 385 nm and emission at 529 nm. In the presence of luciferase, it also generates bioluminescence, emitting green light at approximately 561 nm through a biocompatible enzymatic process [118]. These dual‐mode emissive properties make the CBT–Cys reaction particularly attractive for in vitro and in vivo applications, including biomolecular labeling, biosensing, imaging and detection [109]. Since its initial report in 2010, this reaction has been applied in diverse contexts—such as the site‐specific labeling of proteins, the development of fluorescent and bioluminescent sensors for reactive oxygen species and thiols, tumor imaging through luciferin‐based probes, and the quantitative detection of biomolecules in complex biological samples.

### 5.2 Application in Semi‐Quantitative Analysis

With its wide application in biomolecular labeling, sensing, and detection, the CBT–Cys reaction has also been shown to enable semi‐quantitative analysis by correlating fluorescence or luminescence intensity with the relative abundance of target compounds. Although these approaches do not yield absolute concentrations, the signal intensity provides valuable insight into biological changes or molecular events, often without the need for calibration curves.

**FIGURE 10:**
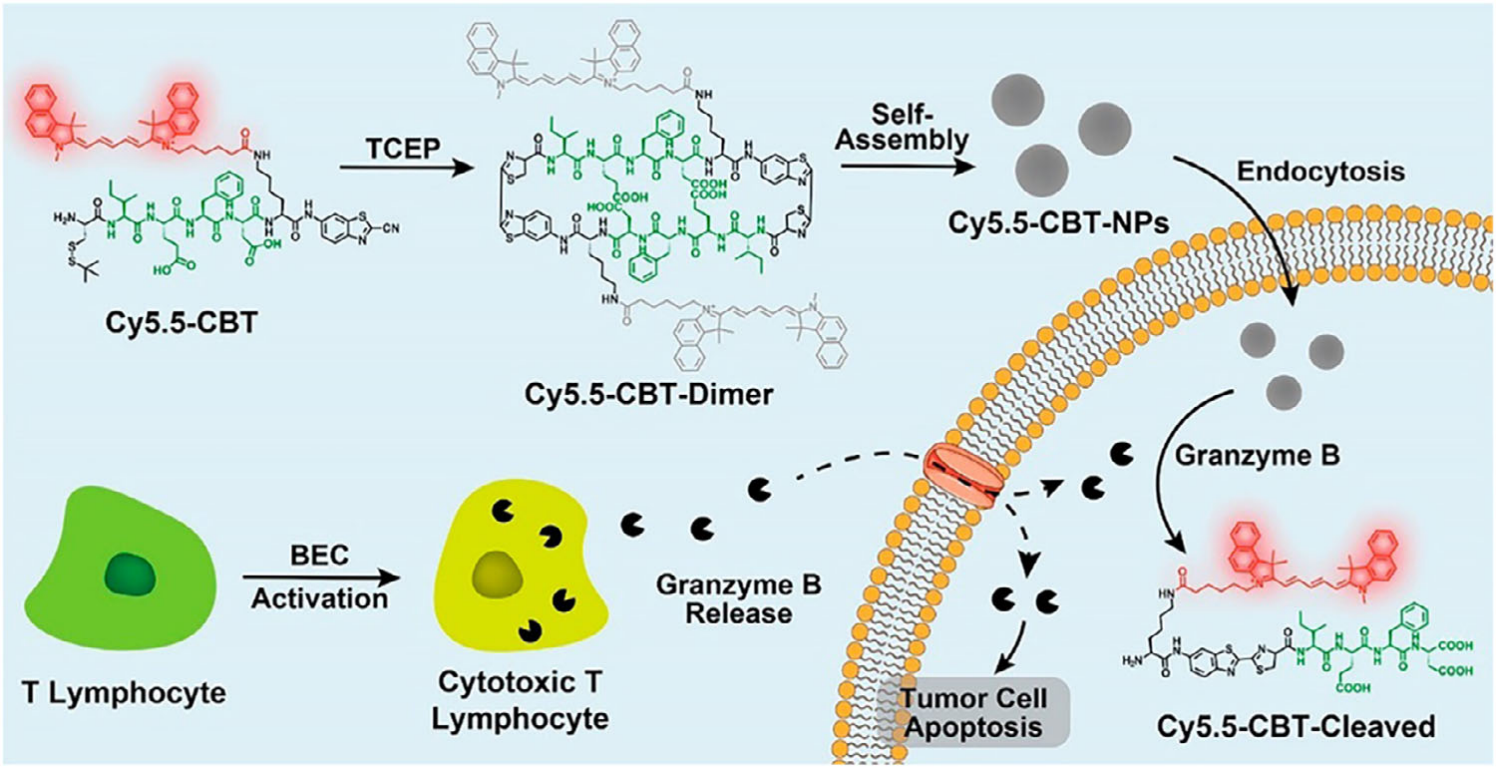
Schematic illustration of fluorescence “dual quenched” Cy5.5‐CBT‐NPs for granzyme B imaging. Reprinted from Reference [120] with permission. Copyright 2022, American Chemical Society.

A straightforward approach for semi‐quantitative analysis using the CBT–Cys reaction involves measuring the fluorescence intensity of its condensation product to estimate the abundance of biothiols or thiol‐containing biomolecules. In 2012, Liang and coworkers [119] reported a method for labeling Cys residues on the eggshell membrane via CBT–Cys condensation. The resulting fluorescence intensity showed a positive correlation with Cys content, though not in a strictly numerical or linear relationship. Later, the same group, Liang and coworkers [120] developed a near‐infrared fluorescence probe, Cys(StBu)‐Ile‐Glu‐Phe‐Asp‐Lys(Cy5.5)‐CBT (Cy5.5–CBT), to construct CBT–Cys‐based nanoparticles (Cy5.5–CBT–NPs) for the imaging of granzyme B, an enzyme associated with cytotoxic T lymphocyte (CTL) activity in cancer immunity (Figure 10). The nanoparticles were formed through CBT–Cys condensation between terminal CBT and Cys groups, resulting in fluorescence quenching. Upon cleavage by granzyme B, the fluorogenic product was released, restoring fluorescence. The fluorescence intensity reflected the level of granzyme B activity and thus enabled evaluation of CTL tumoricidal function.

Fluorescence measurement of the CBT‐Cys condensation products has also been applied in tumor localization and theranostics. In 2016, Ai et al. [121] reported the use of upconversion nanocrystals (UCNs) for tumor localization, leveraging the unique features of the tumor microenvironment. The UCN precursor, sensitive to this environment, could be activated through the removal of protective groups to expose CBT and Cys termini. These termini underwent crosslinking via the CBT–Cys condensation reaction to form fluorescent UCN aggregates in the tumor. The localized fluorescence—detected using confocal microscopy—correlated with tumor site and abundance. Notably, due to the unique physical properties of UCNs, the aggregates were also detectable via photoacoustic imaging, expanding the detection modalities. More recently, Qi et al. [122] reported the application of the CBT–Cys reaction in tumor localization and theranostics using a conjugate of an aggregation‐induced emission luminogen (AIEgen) and peptide (D2P1), together with cyanobenzothiazole‐cysteine (3CBT). These components formed nanoaggregates inside tumor cells via the CBT–Cys reaction. The abundance of these aggregates, visualized through confocal fluorescence imaging, was correlated with signal intensity.

Besides fluorescence measurement, the luminescent properties of the CBT–Cys condensation product also make luminescence‐based detection a practical approach. Godinat et al. [123]. reported a noninvasive bioluminescent imaging method to evaluate protease activity in vivo (Figure 12a). In their method, caged Cys and CBT were injected into luciferase‐transgenic mice. When proteases were active, they cleaved the caged Cys to release free Cys, which then reacted with CBT to form luciferin. In the presence of luciferase, the resulting luminescence was detected and used to evaluate protease activity. The intensity of the luminescence correlated with the enzymatic activity of the protease. Notably, the authors highlighted the versatility of this method, suggesting that it could be adapted for other biomolecules beyond proteases. They also proposed a dual‐imaging concept—enabling the simultaneous visualization of two molecular targets—which further highlights the broad applicability of the CBT–Cys reaction in as a semi quantitative method to analyze the biomolecules.

**FIGURE 11:**
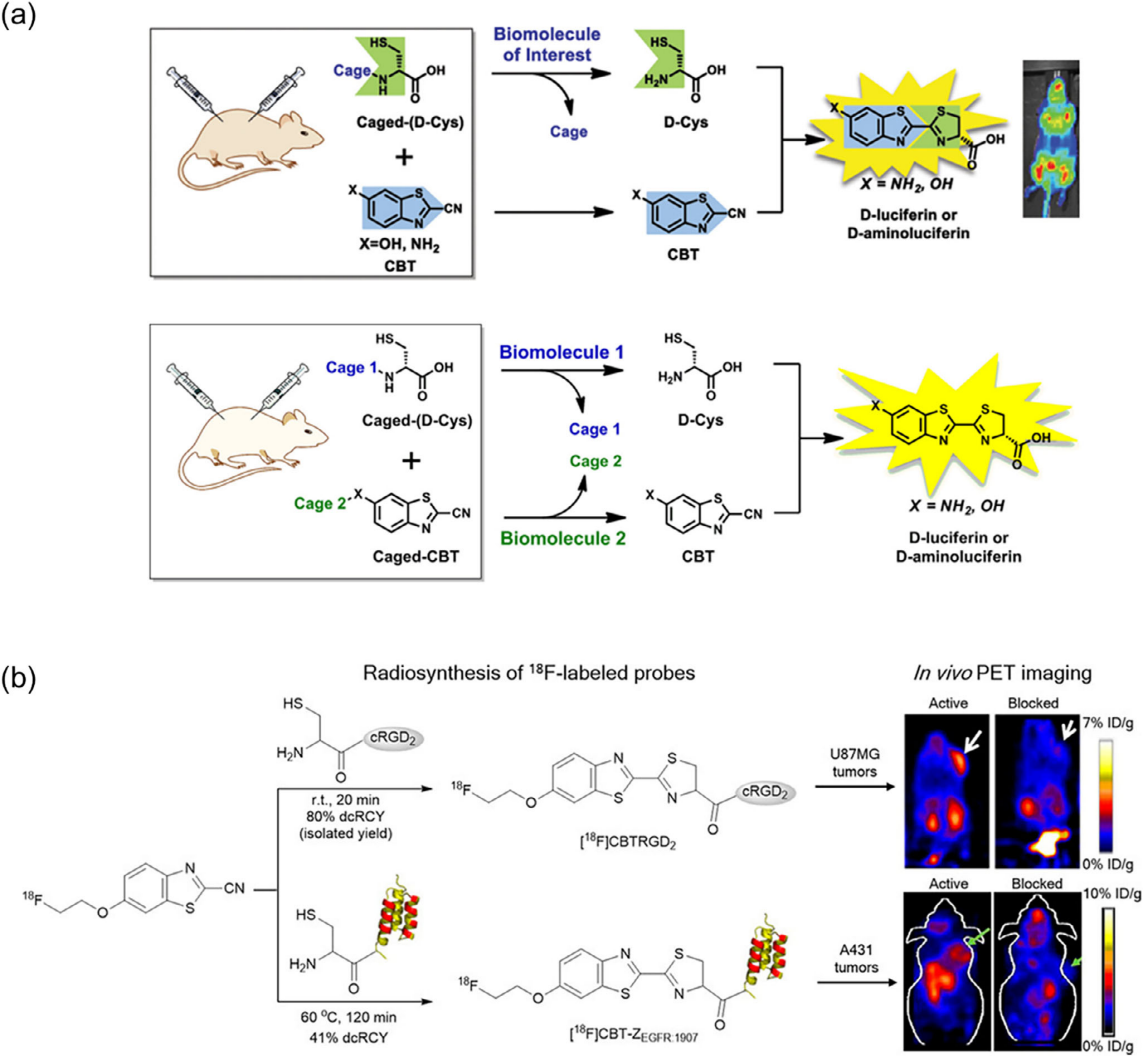
(a) Schematic illustration of the noninvasive bioluminescent imaging method and the dual imaging concept using caged‐CBT and D‐Cys. (b) Schematic illustration of CBT‐Cys reaction in ^18^F labeling. Adapted from References [123] and [113] with permission. Copyright 2013, American Chemical Society and Copyright 2012, American Chemical Society.

Another notable application of the CBT–Cys reaction in semi‐quantitative analysis is its use in PET imaging through ^18^F‐labeling. Chin and coworkers [113] demonstrated that the CBT–Cys condensation reaction is well‐suited for ^18^F‐labeling applications (Figure 11b). In their study, tumor‐targeting proteins were successfully labeled using this reaction, achieving a decay‐corrected radiochemical yield of 12% and a radiochemical purity exceeding 99%. The PET signal reflected the abundance and spatial distribution of the labeled proteins, enabling visualization of the tumor. These results highlight the CBT–Cys reaction's fast kinetics and high chemoselectivity, making it a promising strategy for efficient ^18^F‐labeling in PET imaging.

### 5.3 Application in Quantitative Analysis

The CBT–Cys reaction, characterized by its high reaction rate and excellent selectivity, serves as an ideal candidate for quantitative analysis when integrated with specific analytical techniques. Its rapid kinetics allow for real‐time monitoring of target biomolecules, while the high chemoselectivity minimizes side reactions, ensuring both reproducibility and biocompatibility. These advantages make the CBT–Cys reaction particularly suitable for accurate and reliable quantification in complex biological environments.

Bioluminescence spectroscopy is a powerful and highly sensitive technique for quantitative analysis, capable of detecting analytes at concentrations as low as 10^−12^ M, with a maximum LOD reaching 10^−18^ M under ideal conditions [124]. The luminescent properties of the CBT–Cys condensation products make it feasible to integrate this reaction with bioluminescence spectroscopy for quantitative purposes—although in many bioimaging applications, it is still used in a semi‐quantitative manner.

**FIGURE 12:**
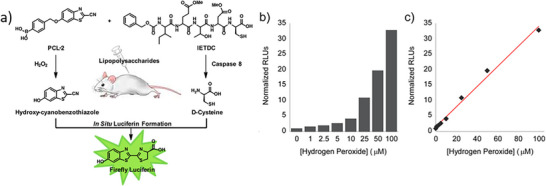
(a) Schematic illustration of the dual analyte luciferin imaging and quantitative analysis of hydrogen peroxide. (b) The normalized RLU and concentration of H_2_O_2_ relationship and (c) line graph representation of (b). Reprinted from Reference [125] with permission. Copyright 2013, American Chemical Society.

In 2013, Van de Bittner et al. [125] first demonstrated a dual‐analyte luciferin imaging method that employed the CBT–Cys reaction for quantitative analysis of biomolecules (Figure 12). They designed a peroxy‐caged luciferin‐2 (PCL‐2), which released CBT and Cys in the presence of hydrogen peroxide (H_2_O_2_) and active caspase‐8. The released precursors underwent in situ CBT–Cys condensation to generate luciferin in luciferase‐transgenic mice, producing a luminescence signal correlated with the levels of H_2_O_2_ and caspase‐8 activity. A calibration curve relating luminescence intensity to the concentration of H_2_O_2_ or caspase‐8 was established. However, due to the complexity of the in vivo environment, the LOD was approximately 0.5 µmol. Later, in 2019, Karatas et al. [126] reported a method to quantify cellular peptide uptake using the CBT–Cys reaction in combination with bioluminescence spectroscopy. In this approach, the target peptide was modified with a disulfide‐linked Cys, which was cleaved by intracellular glutathione (GSH) to release free Cys. The in situ CBT–Cys reaction then generated luciferin, whose luminescence intensity was directly correlated with peptide concentration. A calibration curve was also established to quantify peptide levels from the luminescence signal.

**FIGURE 13:**
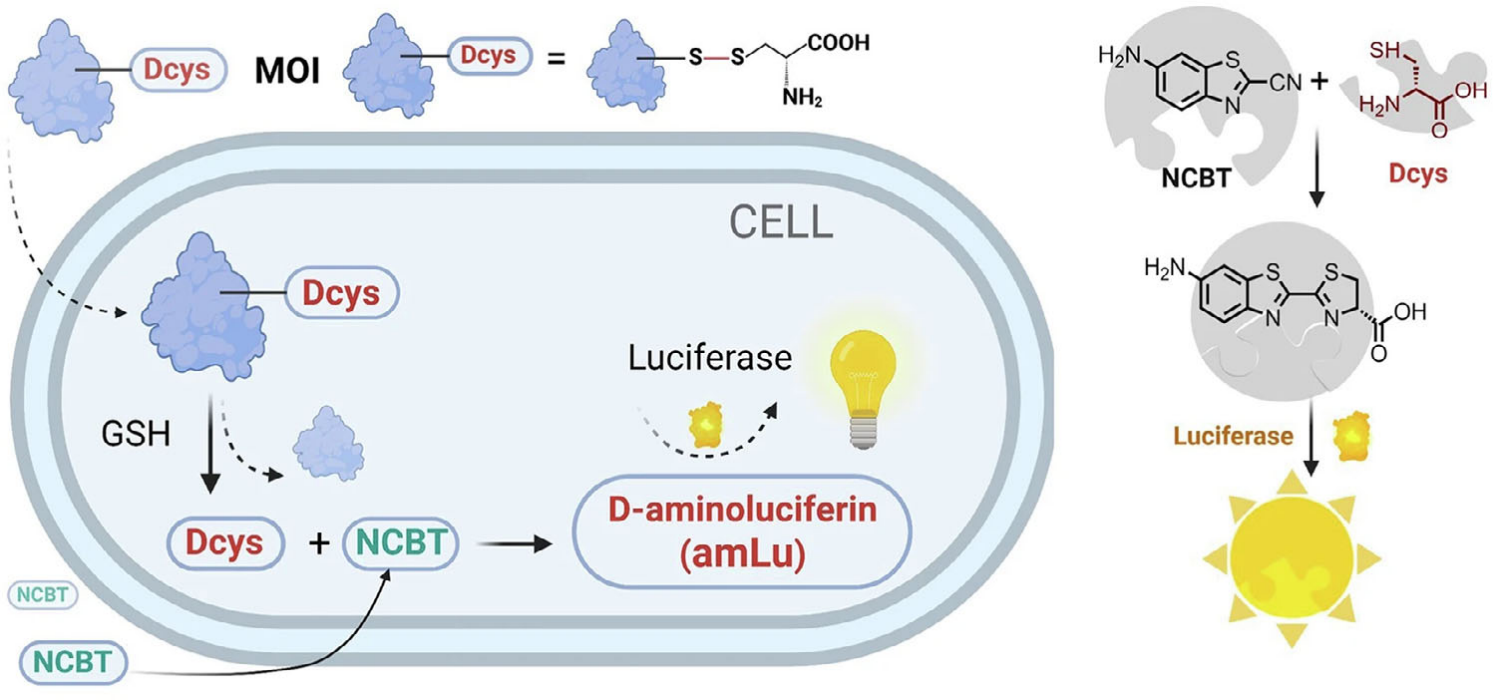
Schematic illustration of the BioLure assay method. Reprinted from Reference [24] with permission. Copyright 2024, The Authors, under CC‐BY 4.0.

More recently, Wang et al. [24] reported a simple and versatile bioorthogonal luminescent reaction‐based assay, termed the BioLure assay, for the quantitative analysis of intracellular delivery of diverse biomolecules (Figure 13). In this method, various target biomolecules, including biothiols, biopolymers and proteins were functionalized with disulfide‐linked Cys and introduced into cells via electroporation. Once inside the cells, intracellular GSH reduced the disulfide bond, releasing free Cys. The free Cysc subsequently reacted with CBT to form luciferin, producing a luminescent signal proportional to the concentration of the delivered biomolecules. A calibration curve correlating luminescence intensity with CBT and Cys concentrations was established to evaluate the efficiency of biomolecules delivery with electroporation method. This approach demonstrated the broad applicability of the BioLume assay for tracking and quantifying a wide range of intracellular targets in a biocompatible and label‐efficient manner.

**FIGURE 14:**
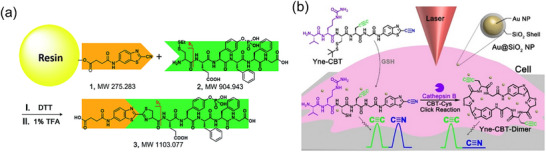
(a) Schematic illustration of using solid phase CBT derivative **1** to fish N‐terminal Cys peptide **2**. (b) Schematic illustration of the CTSB‐triggered CBT‐Cys click reaction for quantitative SHINERS imaging of the CTSB activity in a single cell. Adapted from References [112] and [130] with permission. Copyright 2017, RSC under CC‐BY 3.0 and Copyright 2024, American Chemical Society.

The CBT–Cys reaction has also been integrated with standard quantitative analytical techniques, including UV–vis absorption spectroscopy, fluorescence spectroscopy, and MS, to enable accurate quantification of biomolecules. Yuan et al. [127]. reported a CBT‐Cys(SEt) probe for selective GSH quantification using UV absorption spectroscopy both in vitro and in HepG2 liver cancer cells. The molecule contains a CBT terminal and a disulfide‐linked Cys terminal. In the presence of GSH, the disulfide bond is reduced to release free Cys, which condenses with CBT to form nanorings showing absorbance at 380 nm. A calibration curve of the GSH concentration was established in vitro, with a LOD of 1 µM. Miao et al. [128]. reported another approach using a self‐quenched fluorescent probe to quantify various biothiols both in vitro and in living cells via fluorescence spectroscopy. Upon reaction with biothiols, the CBT–Cys condensation product produces a fluorescence signal, which correlates with biothiol concentration. The method demonstrated a LOD of 0.246 µM for GSH. Zheng et al. [112] developed a method using solid‐phase CBT to capture peptides with N‐terminal cysteine (Figure 14a). In this approach, CBT was immobilized on resin via a succinic anhydride linker. A custom‐built microreactor coupled with MS enabled real‐time monitoring and mechanistic investigation of the CBT–Cys reaction. Due to the high sensitivity and specificity of MS, the captured peptides were accurately identified by TOF‐MS and quantitatively analyzed based on signal intensity. A calibration curve correlating peptide amount with MS intensity was established, enabling precise quantitative analysis.

The CBT–Cys reaction has also been integrated with Raman spectroscopy, a functionality‐sensitive technique, for biomolecule quantification. In 2017, Li et al. [129]. developed a Raman probe consisting of a CBT moiety conjugated to poly(ethylene glycol)‐modified 60 nm gold nanostars. The probe featured a cyano group that produced a distinct Raman peak. Upon reaction with intracellular biothiols, the CBT–Cys condensation eliminated the cyano group signal, allowing indirect quantification of biothiol concentrations. Calibration curves for three different biothiols were established, and intracellular concentrations were successfully measured. More recently, Wang et al. [130]. reported a self‐referenced Raman probe, Val‐Cit‐Cys(StBu)‐Pra‐Gly‐CBT (Yne–CBT), for quantifying cathepsin B activity. In the presence of the enzyme, the probe underwent a CBT–Cys reaction to form a dimer lacking the cyano group (Figure 14b). An alkyne group on the probe served as an internal standard, improving quantification accuracy. A calibration curve between relative Raman intensity and cathepsin B concentration was established, with a LOD of 61.4 U·L^−^^1^.

## 6 Strengths, Limitations, and Suitability of Bioorthogonal Reactions for Quantification

We summarize the kinetics and applications of the four bioorthogonal reactions discussed above in Table 1. From the perspective of detection methods, in semi‐quantitative analysis, common detection methods include confocal fluorescence microscopy or PET imaging. Fluorescence microscopy is compatible with all four bioorthogonal reactions, either through the intrinsic fluorescence of certain probes or through conjugation with suitable fluorescent tags. PET imaging is likewise applicable to all these reactions, with a notable trend that faster reactions are more frequently used with various isotopes. This is because rapid kinetics ensure efficient labeling and detection within the short half‐lives of radioactive isotopes [131]. Luminescence imaging is typically applied with the CBT–Cys reaction, although it can also be used with Staudinger ligation when luciferin is appropriately modified. Western blotting is generally used to assess relative content, particularly when the substrate lacks an intrinsic fluorescence signal. It is often combined with biotin labeling to enable further detection through confocal fluorescence microscopy. In quantitative analysis, high‐precision detection techniques such as fluorescence and luminescence spectroscopy are used for obtaining absolute amount of target molecules. Luminescence spectroscopy exhibits a lower intrinsic LOD than fluorescence spectroscopy, being capable of detecting analytes at concentrations as low as 10^−12^ M and, under ideal conditions, reaching 10^−18^ M [124]. However, in practical applications, the LODs achieved by both methods are typically in the nanomolar range, reflecting the limitations imposed by the complex biological environment. Two notable exceptions demonstrate much higher sensitivity: one electrochemical method reported a LOD in the attomolar range, and a fluorescence spectroscopy approach achieved a LOD in the femtomolar range. It is important to note that reported LODs often vary in units and calculation methods, making direct comparisons challenging. For instance, some studies report LODs in terms of amol per cell or ng/mL, which may not be directly comparable to molar concentrations.

**TABLE 1:** Summary of the catalyst‐free bioorthogonal reactions and their applications in the semi‐quantitative and quantitative analysis of biomolecules.

Reaction	Semi‐quantitative			Quantitative			
	Biomolecules	Techniques	Ref.	Biomolecules	Techniques	Limit of detection (LOD)	Ref.
Staudinger ligation (Second‐order Rate constant k_2nd_ = 10^−3^ M^−1^ s^−1^)	Fatty‐acylated proteins	Western blot	[37]	Nitroxyl	Fluorescence spectroscopy	4.3 × 10^−^^8^ M	[47]
	Glycans	Confocal fluorescence microscopy	[38]	Nucleic acid	Fluorescence spectroscopy	1.3 × 10^−^^1^⁵ M	[48]
	Nucleic acid	Western blot	[39]	Glucose	Luminescence spectroscopy	9.6 × 10^−^^8^ M	[22]
	DNA	Spectrofluorometer	[41]	Peptidoglycan	Luminescence spectroscopy	Standard curve reported. No LOD	[49]
	Glycans	Fluorescence microscopy	[42]				
	Proteins	Confocal fluorescence microscopy	[43]				
	Glycans	CCD camera photon detection	[44]				
	Proteins	PET imaging	[46]				
SPAAC (Second‐order Rate constant k_2nd_ = 0.01‐60 M^−1^ s^−1^)	Glycans with sialic acids residuals	Confocal fluorescence microscopy	[54]	Glycans	Flow cytometry and confocal microscopy	Standard curve reported. No LOD	[69]
	Sialic acids	Confocal fluorescence microscopy	[61]	Tumor markers (proteins)	Luminescence spectroscopy	0.085 ng·mL^−^^1^ and 0.044 ng·mL^−^^1^ for two proteins	[70]
	Peptide nucleic acids	Confocal fluorescence microscopy	[39]	Peptides	Mass spectrometry and Confocal fluorescence microscopy	0.0002 amol per cell	[71]
	Proteins	Western blot	[62]	Peptides	ToF‐SIMS	1.5× 10^−^^7^ M and 6.1 × 10^−^^7^ M	[72]
	DNAs	Western blot	[63]	Bruton's tyrosine kinase (proteins)	Mass spectrometry	Only the absolute quantification value 61.28 ng per 10⁶ cells reported.	[73]
	G protein‐coupled receptors	Western blot	[64]	miRNA biomarkers	Electrochemical signals	7.5 × 10^−^^17^ M	[23]
	Nucleotides	Fluorescence microscopy	[65]				
	Bombesin (peptides)	PET imaging	[66]				
	α_v_β_6_ integrin targeting peptide	PET/CT imaging	[21]				
	Epidermal growth factor receptor	PET/CT imaging	[67]				
	Oligonucleotide	PET imaging	[68]				
IEDDA (Second‐order Rate constant k_2nd_ = 1–10^6^ M^−1^ s^−1^)	Antibodies	Confocal fluorescence microscopy	[87]	Antibody ITGB1 and TGM1	Immuno‐PCR	0.095 and 0.094 for ITGB1 and TGM1	[104]
	IFITM3 (proteins)	Confocal fluorescence microscopy	[88]	ATP	Fluorescence ratiometric techniques	0.0354 mM and 0.0121 mM	[105]
	Proteins	Confocal fluorescence microscopy	[89]				
	Antibodies	Confocal fluorescence microscopy	[90]				
	Fatty acids, sugars, and amino acids	Confocal fluorescence microscopy and Fluorescence spectroscopy	[91]				
	Proteins	Confocal fluorescence microscopy and western blotting	[92]				
	Peptide	Confocal fluorescence microscopy	[93]				
	Antibodies	PET imaging	[94]				
	Epidermal growth factor receptor 3	PET imaging	[95]				
	Nucleic acids	PET imaging	[96]				
	Biopolymers	PET imaging	[97]				
	Vascular endothelial growth factor	PET imaging	[98]				
	Nanoparticles	PET imaging	[99]				
	Antibody U36	PET imaging	[100]				
	bombesin antagonist PEG_4_‐AR	Cerenkov luminescence imaging	[102]				
	Antibody AR9.6	Cerenkov luminescence imaging	[103]				
CBT‐Cys (Second‐order Rate constant k_2nd_ = 1–10 M^−1^ s^−1^)	Cys residue	Confocal fluorescence microscopy	[119]	H_2_O_2_ and caspase‐8 activity	Luminescence spectroscopy	0.5 µmol	[125]
	Granzyme B (proteins)	Confocal fluorescence microscopy	[120]	Peptide	Luminescence spectroscopy	Standard curve reported. No LOD	[126]
	Upconversion nanocrystals in tumor	Confocal fluorescence microscopy	[121]	Biothiols, biopolymers and proteins	Luminescence spectroscopy	Standard curve reported. No LOD	[24]
	Peptide	Confocal fluorescence microscopy	[122]	Glutathione	UV absorption spectroscopy	1 µM	[127]
	Caspase 3/7	Luminescence imaging	[123]	Biothiols	fluorescence spectroscopy	0.246 µM	[128]
	Tumor‐targeting proteins	PET imaging	[113]	Peptides	Mass spectrometry	Standard curve reported. No LOD	[112]
				Biothiols	Raman spectroscopy	Standard curve reported. No LOD	[129]
				Cathepsin B activity	Raman spectroscopy	61.4 U·L^−^^1^	[130]

From the reaction perspective, Staudinger ligation, as the first reported bioorthogonal reaction, has been extensively studied for over two decades. However, its slow reaction kinetics and susceptibility to side reactions have limited its application in both semi‐quantitative and quantitative analyses. In particular, for quantitative applications, the Staudinger reaction often requires additional strategies, such as signal amplification, to achieve acceptable LODs, which limits its practical utility compared to faster bioorthogonal reactions [48]. In semi‐quantitative applications, it has primarily been used to label large biomolecules such as proteins, glycans, and nucleic acids, as azide groups can be readily introduced into these targets through metabolic labeling and with good stability. For quantitative analysis, its use has been more focused on small molecules and nucleic acids. Larger biomolecules, such as peptidoglycan, are more challenging to quantify accurately; for example, peptidoglycan quantification has only been reported with a limited linear range and without a defined LOD. Notably, a distinct advantage of the nontraceless Staudinger ligation is its ability to directly release a caged molecule upon reaction. In contrast, other bioorthogonal reactions typically require additional functional groups such as disulfide bonds for triggered release. This unique property makes Staudinger ligation particularly suitable for releasing small molecules in applications that demand high detection accuracy. Overall, the Staudinger ligation reaction is suitable for semi‐quantitative detection of glycan‐related biomolecules in fixed or stabilized cell samples, or under in vitro conditions, using fluorescence microscopy. In vivo, it is likely applicable to long‐lived proteins that remain stable over extended periods.

The SPAAC reaction offers significantly faster kinetics compared to Staudinger ligation and can achieve second‐order rate constants greater than 1 M^−^^1^ s^−^^1^ with appropriate modification of the substrates. In semi‐quantitative analysis, SPAAC is commonly used for labeling large biomolecules, similar to Staudinger ligation, due to the simple incorporation of azide groups into proteins, nucleic acids, and glycans. However, it can also be used for small molecules such as sialic acid which can incorporate with the azide group. In quantitative analysis, SPAAC is also predominantly applied to large biomolecules. However, as previously mentioned, quantitative studies often lack a clearly reported LOD. The few available LOD values are typically obtained using MS, which is well‐suited for quantifying macromolecules. Remarkably, one study achieved an ultralow LOD using an electrochemical detection method, demonstrating the potential of SPAAC for highly sensitive quantitative analysis when paired with advanced detection techniques. Therefore, considering the fast kinetics and good stability of strained cyclic alkynes and azide groups, SPAAC is suitable for semi‐quantitative detection of glycan‐related biomolecules in fixed cell samples and its shorter reaction half‐life further enables its application in quantitative analysis and in vivo dynamic studies with a broader scope of biomolecules.

The IEDDA reaction exhibits the fastest kinetics among the four bioorthogonal reactions discussed. Combined with the unique reactivity of Tz chemistry, it has been applied to a broad range of targets from large biomolecules to small molecules in semi‐quantitative analyses. This highlights the value of the IEDDA reaction as a powerful tool for semi‐quantification. Considering current labeling strategies, IEDDA handles can be readily introduced into proteins via genetic code expansion, making this reaction particularly suitable for semi‐quantitative and quantitative analysis of proteins in vivo, with potential for real‐time quantification of biomolecules. However, its application in fully quantitative analysis remains limited. One possible contributing factor could be the relatively large molecular size of both Tz and dienophile partners such as TCO, which may influence assay performance and complicate precise quantification [132]. Another reason is the limited stability of Tz with some basic condition or the redox reagent. In the few available examples, the reported LODs are relatively high, typically in the micromolar range, though this may also reflect the limited number of studies rather than an inherent limitation of the reaction.

The CBT–Cys reaction, derived from a naturally occurring process, also exhibits relatively fast kinetics. In semi‐quantitative analysis, it can be applied to large biomolecules as well as cysteine residues, which closely resemble the cysteine‐based substrate. For quantitative analysis, the reaction has been used to detect both small molecules such as biothiols and larger biomolecules like enzymes and nucleic acids. One notable feature is that the reaction produces the luciferin, substrate of bioluminescence reaction, making it well‐suited for integration with luminescence spectroscopy. However, it has not yet achieved ultra‐low LOD expected from luminescence spectroscopy (e.g., in the attomolar range), likely due to the complexity of the system. The natural thiol group in cysteine facilitates caging and release mechanisms, allowing the reaction to target large biomolecules and enabling its compatibility with various techniques such as Raman spectroscopy, UV absorption spectroscopy, and MS. These properties highlight CBT–Cys as an important tool for the quantitative analysis of biomolecules. Overall, the endogenous presence of D‐Cys and the simplicity of disulfide chemistry make the CBT–Cys reaction suitable for both semi‐quantitative and quantitative analysis of thiol‐containing biomolecules in vivo. Moreover, the high sensitivity of luminescence spectroscopy, combined with the fast reaction kinetics, also enables its use for real‐time kinetic monitoring in living systems.

## 7 Summary and Outlook

Catalyst‐free bioorthogonal reactions have emerged as indispensable tools in the labeling and quantification of biomolecules due to their biocompatibility, selectivity, and operational simplicity under physiological conditions. In this review, we examined four representative reactions—Staudinger ligation, SPAAC, IEDDA, and CBT–Cys condensation—focusing on their kinetics, scope of biomolecular targets, detection strategies, and quantitative performance.

Each reaction offers unique advantages. Staudinger ligation, despite its slow kinetics, remains valuable for the high stability of its azide handle and well‐established labeling strategies. SPAAC provides improved kinetics and broad applicability, especially with specific substrate modifications. IEDDA stands out for its extremely fast kinetics and wide substrate scope in semi‐quantitative analysis, although challenges in precise quantification remain due to bulky reactants and limited handle stability. The CBT–Cys reaction demonstrates versatility across biomolecular types and detection techniques, particularly through its ability to produce luciferin for luminescent assays.

For further development, several key challenges and opportunities should be addressed to advance this field. First, as bioorthogonal chemistry is still in its early stage of application for biomolecule quantification, establishing standardized reporting practices, such as unified units for LOD, defined linear ranges, and clearly specified measurement conditions, is essential. Such standardization will enable meaningful comparison across studies and facilitate systematic progress. Second, improving the sensitivity and reproducibility of quantitative detection remains a major goal. This can be achieved by designing more efficient probes, minimizing background signals, and integrating bioorthogonal chemistry with advanced analytical techniques. Enhancing chemoselectivity is also critical for achieving higher sensitivity and reproducibility in complex biological environments. Third, efforts should continue to focus on accelerating the kinetics of bioorthogonal reactions. Faster reaction rates not only improve overall efficiency but also enhance the applicability of these methods in dynamic biological systems [12, 17]. In contrast, slow reactions often require additional strategies, such as incorporating amplification designs, which introduce further challenges and complexity.

Recently, new bioorthogonal technologies such as cysteine‐to‐tetrazine conjugation and cleavable IEDDA reactions have emerged [133, 134]. Both approaches enable controlled release of targeted molecules within cells. In particular, cysteine‐to‐tetrazine conjugation provides chemoselective labeling of cysteine even in the presence of a large excess of other thiols. Although these strategies have not yet been applied to biomolecule quantification, they hold significant potential for developing more sensitive and accurate quantitative methods.

Overall, the continued development and refinement of catalyst‐free bioorthogonal reactions will contribute to more precise and accessible biomolecular quantification, with far‐reaching implications for both fundamental research and translational applications.

## Conflicts of Interest

The authors declare no conflict of interest.

## Data Availability

This is a review paper without original data to share.
